# Hydrophobic and Superhydrophobic Coatings: Materials, Fabrication Strategies, and Durability Challenges

**DOI:** 10.3390/ijms27167323

**Published:** 2026-08-16

**Authors:** Natalia A. Shapagina, Vladimir V. Dushik

**Affiliations:** Frumkin Institute of Physical Chemistry and Electrochemistry Russian Academy of Sciences, Leninsky Prospect 31-4, 119071 Moscow, Russia; v.dushik@gmail.com

**Keywords:** hydrophobic coatings, superhydrophobic coatings, wettability, surface modification, durability, self-cleaning surfaces, corrosion protection, sustainable materials

## Abstract

Hydrophobic and superhydrophobic coatings have attracted considerable attention due to their ability to provide water repellency, self-cleaning, anti-corrosion, anti-icing, and anti-fouling properties, making them promising for a wide range of industrial applications. This review summarizes recent advances in the development of hydrophobic and superhydrophobic coatings, with particular emphasis on wetting mechanisms, material selection, coating formation approaches, durability issues, commercial implementation, and environmental aspects. The analysis examines the principal classes of materials used for coating fabrication, including polymeric materials, inorganic compounds, and composite systems. The mechanisms responsible for the formation of hydrophobic and superhydrophobic surfaces are discussed in terms of surface chemistry modification and hierarchical roughness generation. Attention is devoted to factors limiting long-term performance, such as mechanical wear, chemical degradation, ultraviolet exposure, climatic effects, hydrodynamic erosion, and adhesion-related failures, as well as to current strategies for improving durability. Commercially available technologies and their application areas are reviewed, and the environmental challenges associated with fluorinated compounds are considered. The analysis demonstrates that the combination of controlled surface morphology and reduced surface energy remains an effective approach for achieving durable hydrophobicity, with optimized coating systems reaching contact angles of 160–170° and retaining superhydrophobic properties for more than 500 h under demanding operating conditions. Future developments are expected to focus on environmentally friendly, multifunctional, and long-lasting coating systems.

## 1. Introduction

### 1.1. Fundamental Concepts and Definitions

The phenomenon of superhydrophobicity, first observed in nature on lotus leaves and commonly referred to as the “lotus effect,” has attracted considerable scientific interest over the past decades owing to its unique combination of water-repellent and self-cleaning properties. Advances in the fabrication of micro- and nanostructured surfaces have enabled the successful replication of this effect under laboratory conditions, leading to the development of a wide variety of hydrophobic and superhydrophobic coatings based on diverse material systems. At present, research in this field has extended beyond fundamental studies and is increasingly focused on addressing practical engineering challenges. Hydrophobic (HP) and superhydrophobic (SHP) coatings have found applications in construction, transportation, energy systems, microelectronics, the oil and gas industry, and numerous other industrial sectors. The growing number of commercial products and the expanding range of practical applications indicate a gradual transition of these technologies from laboratory-scale research to industrial implementation. In this context, a comprehensive assessment of the current state of the field, including the materials employed, coating formation mechanisms, durability challenges, commercial solutions, and environmental aspects of their application, is both timely and necessary [1,2,3,4,5,6,7,8,9,10,11,12,13,14,15,16,17,18,19,20,21,22,23,24,25].

To facilitate a fundamental understanding of hydrophobic and superhydrophobic coatings, several key terms and definitions commonly used in this field should be introduced:

- Wettability—the tendency of a liquid to establish contact with and spread over a solid surface, determined by the balance of adhesive interactions between the liquid and the solid and cohesive forces within the liquid itself [3,26,27];

- Static water contact angle (WCA)—the angle formed between the tangent to a liquid droplet and the solid surface at the three-phase contact line under equilibrium conditions. A surface is generally classified as hydrophobic when the water contact angle exceeds 90°, and as superhydrophobic when it exceeds 150° [19,27,28,29,30];

- Hydrophobicity—the property of a material or surface characterized by low affinity for water, resulting in limited wetting and relatively high water contact angles [3,4,7,8,9,10];

- Superhydrophobicity—a surface state characterized by extremely low wettability, typically defined by a static water contact angle greater than 150° and a sliding (or roll-off) angle below 10°, enabling water droplets to remain nearly spherical and roll off the surface with minimal adhesion [1,2,5,6,15,16,17,18,19];

- Surface roughness—the deviation of a real surface from an ideal geometrically smooth surface, expressed by the presence of surface asperities and texture over different length scales. In combination with low surface energy, hierarchical roughness is a key factor governing superhydrophobic behavior by promoting composite solid–air–liquid interfaces [1,2,3,8,26];

- Hydrophobizing agent—a substance or chemical treatment applied to a surface to reduce its surface free energy and increase water repellency by introducing hydrophobic functional groups or forming a low-energy surface layer [31,32,33];

- Anti-icing properties—the ability of a surface or coating to suppress, delay, or mitigate ice nucleation, ice accumulation, and/or ice adhesion under icing conditions [22,25,33,34];

- Corrosion resistance—the ability of a material or protective coating to resist chemical or electrochemical degradation caused by exposure to aggressive environments, thereby preserving the integrity and functionality of the substrate [14,35,36,37];

- Liquid droplet erosion (water droplet erosion)—the progressive degradation of a material surface caused by repeated high-velocity impacts of liquid droplets, leading to material removal, surface fatigue, and deterioration of functional properties [38,39,40];

- Biofouling—the undesirable accumulation of microorganisms, plants, algae, or animals on a wetted surface, often resulting in deterioration of surface performance and functionality [41,42,43,44,45];

- Antifouling coatings—coatings designed to prevent or minimize the adhesion and accumulation of biological organisms, contaminants, or other unwanted deposits on a surface. In superhydrophobic coatings, antifouling performance is primarily achieved by minimizing the contact area and adhesion between the surface and foulants rather than by biocidal action [44,45,46,47].

Several wetting models have been proposed to describe the interaction of liquids with solid surfaces [48,49,50]:

- Young’s model: This model applies to ideally smooth, chemically homogeneous surfaces and is based on the equilibrium of interfacial forces acting at the three-phase contact line. According to Young’s theory, the water contact angle is determined by Equation (1):

(1) $$\cos\theta =\;\frac{\gamma_{sv}-\gamma_{sl}}{\gamma_{lv}},$$

where θ is water contact angle; $\gamma_{sv}$ is the solid–vapor interfacial energy; $\gamma_{sl}$ is the solid–liquid interfacial energy; and $\gamma_{lv}$ is the liquid–vapor interfacial energy.

- Wenzel’s model: This model describes wetting on rough, chemically homogeneous surfaces in which the liquid fully penetrates the surface asperities (homogeneous wetting regime). In this case, the apparent (effective) contact angle, *θ*′, is related to the intrinsic contact angle, *θ*, through the surface roughness factor *r*, defined as the ratio of the real to the projected surface area. The relationship is given by Wenzel’s Equation (2):

(2) $$\cos\theta^{\prime }=\;r\;\times \;\cos\theta ,$$

where $\theta^{\prime }$ is apparent contact angle and r is the ratio of the actual surface area to its projected area.

- Cassie–Baxter model: This model describes wetting on heterogeneous rough surfaces, where a liquid droplet contacts only the tops of surface asperities while air is trapped within the surface texture (heterogeneous wetting regime). It is typically applied to composite interfaces and accounts for the presence of air pockets. The apparent contact angle is given by Equation (3):

(3) $$\cos\theta^{\prime }=\;f_{s}(\cos\theta_{s}+1)\;-1\;,$$

where $f_{s}$ is the fraction of solid surface in contact with the liquid and $\theta_{s}$ is the intrinsic contact angle of the solid phase.

**Figure 1:**
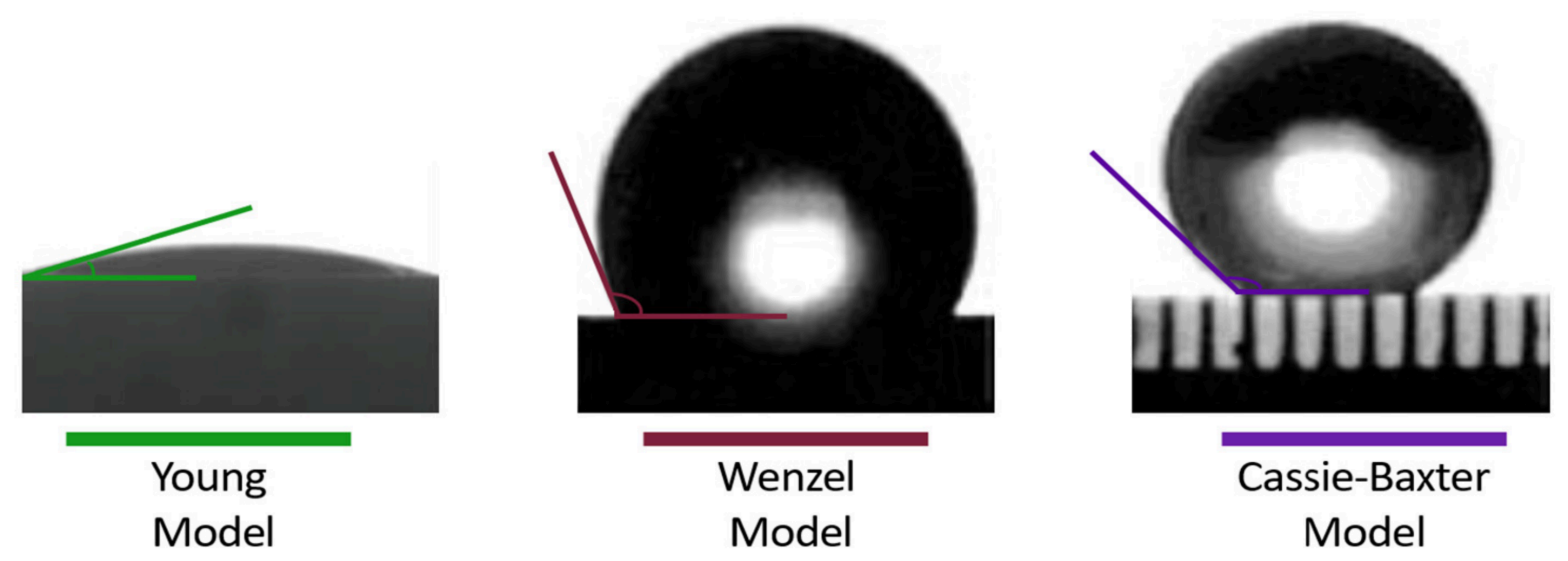
Liquid droplet on solid surfaces, representing the Young, the Wenzel and the Cassie-Baxter models.

A schematic representation of the discussed wetting models is presented in Figure 1.

### 1.2. Classification and Key Parameters of Hydrophobic and Superhydrophobic Materials

Hydrophobic (HP) coatings are defined as coatings capable of repelling water and preventing moisture accumulation on a surface. The primary characteristic of hydrophobicity is a water contact angle (WCA) exceeding 90°. The key features of HP coatings include low surface energy of the material, the formation of nearly spherical water droplets, minimal interaction between water and the surface, and the presence of surface microstructure that enhances the hydrophobic effect [3,4,7,8,9,10,11,12,23,34].

Superhydrophobic (SHP) coatings represent an advanced class of hydrophobic materials, characterized by a water contact angle exceeding 150° and a sliding angle below 10°. The key features of SHP coatings include extremely weak water–surface interactions, spontaneous droplet rolling-off at minimal inclination, self-cleaning behavior, corrosion and anti-icing protection, as well as antibacterial properties [35,36,37,41,42,43,44,46,47,51].

Despite the significant research interest in superhydrophobic coatings, their practical implementation is not always driven by the need to solve a single specific problem. For many functionalities associated with superhydrophobicity, alternative and often more cost-effective solutions already exist. For example, corrosion protection can be achieved using conventional inhibitors and protective coatings, anti-icing performance can be provided by de-icing agents or heating systems, and surface cleanliness can be maintained through various cleaning and washing methods. However, the key advantage of superhydrophobic coatings lies in their ability to simultaneously provide multiple functional properties, including water repellency, self-cleaning, anti-icing, corrosion protection, and antifouling performance. This multifunctionality makes superhydrophobic coatings promising candidates for a wide range of engineering and industrial applications. The most important properties of HP and SHP coatings are summarized in Table 1.

When designing and implementing hydrophobic and superhydrophobic coatings, it is important to consider the factors affecting their long-term durability, including mechanical strength, chemical resistance, stability under temperature fluctuations, resistance to ultraviolet (UV) radiation, and other service conditions.

A considerable number of review articles have been devoted to surface modification strategies for imparting hydrophobic and superhydrophobic properties [1,2,58,59,60]. These reviews have addressed the fundamental aspects of wetting, methods for fabricating textured surfaces, the characteristics of specific material classes, mechanical properties, and practical applications. However, the rapid growth in research activity and the gradual transition of hydrophobization technologies from laboratory-scale developments to industrial implementation necessitate the continuous updating and systematization of existing knowledge, with particular emphasis on technological solutions possessing high practical potential.

The present review aims to summarize the most widely investigated and technologically mature approaches for the fabrication of hydrophobic and superhydrophobic coatings. Particular attention is given to strategies based on an integrated approach combining the deposition of a functional coating layer with subsequent surface modification to achieve hydrophobic or superhydrophobic behavior. The review examines the principal classes of materials used for hydrophobic and superhydrophobic coatings, the mechanisms governing their functional properties, contemporary fabrication and surface modification methods, as well as durability-related challenges and approaches for overcoming them.

In addition, commercially available technologies, the current market status of hydrophobic and superhydrophobic coatings, and the environmental aspects associated with their development and application are analyzed. The presented analysis provides an assessment of the current state of the technology, identifies existing limitations, and highlights the most promising directions for future research and industrial implementation.

## 2. Fabrication Methods for Hydrophobic and Superhydrophobic Coatings: Processing, Materials, and Performance Evaluation

Efficient technologies have been developed for the fabrication of hydrophobic and superhydrophobic coatings, each characterized by its own specific features, advantages, and limitations. A key factor in the formation of hydrophobic and superhydrophobic surfaces is the combination of two requirements: reduction in the surface energy of the material and the creation of an appropriate surface roughness. The latter can be achieved through the use of functional fillers, intrinsic structural features of the coating, or the specific nature of the deposition process itself.

The selection of an optimal fabrication method depends on the type of substrate (metal, glass, ceramic, polymer, etc.), the required functional properties of the coating (wear resistance, electrical conductivity, transparency, etc.), the geometry of the component, operating conditions, economic considerations, and process scalability. The key aspects of the most widely used technologies for the fabrication of hydrophobic and superhydrophobic coatings are briefly discussed below.

### 2.1. The Main Methods of Relief Formation and Hierarchical Roughness

Hierarchical roughness is a key prerequisite for the fabrication of hydrophobic and superhydrophobic coatings, as the surface morphology determines the ability to retain an air layer and provide high contact angle values. In most cases, achieving a superhydrophobic state (contact angle ≥ 150°) requires a combination of surface structuring followed by a reduction in surface energy through chemical modification. The main methods for creating surface relief and the characteristic quantitative parameters used to evaluate the resulting morphology and hydrophobic properties are summarized below:

- Etching enables the formation of microstructures through the selective dissolution of the substrate material. Quantitative characterization of the surface relief is performed using parameters such as the arithmetic mean roughness (R_a_) and the arithmetic mean surface height (Sa). For example, electrochemical etching of steel in a cobalt sulfate solution results in R_a_ ≈ 35.4 nm and S_a_ ≈ 28.4 nm, while subsequent chemical modification yields a contact angle of 144°. The depth of microcavities and the presence of a multilevel (hierarchical) structure (a combination of micro- and nanoscale features) are also of great importance, since such a surface relief most effectively traps air and promotes the development of superhydrophobicity [61,62];

- Anodization enables the formation of porous oxide layers, particularly on aluminum and titanium substrates. The key quantitative characteristics in this case are the pore diameter and oxide layer thickness. It has been established that surface roughness (including the R_a_ parameter) can be deliberately controlled by varying the process conditions, including the electrolyte composition (e.g., a mixture of H_3_PO_4_ and HF) and the treatment duration. Increasing the anodization time results in an increase in Ra. However, excessive roughness may adversely affect other functional properties of the material, for example, its interaction with biological media in the case of implants [63,64];

- Laser treatment provides the possibility of precisely producing microtextures and multilevel hierarchical structures. The morphology of the surface relief is described using the average surface height (S_a_), the maximum surface height (S_z_), and the texture aspect ratio (S_tr_), defined as the ratio of the average width of surface features to their average height. A linear correlation between laser fluence (energy per unit area) and the increase in Sa has been experimentally demonstrated, which, in turn, positively affects the contact angle. Another important technological parameter is the uniformity of the texture, since a non-uniform surface relief may lead to instability of the hydrophobic effect [65,66];

- Lithography provides highly accurate replication of predefined surface microrelief and is more commonly employed for research purposes. Quantitative evaluation mainly focuses on the geometric parameters of the structures, including the feature width, spacing, and relief depth. This method enables the fabrication of well-controlled micro- and nanostructures, and published studies provide a detailed analysis of the influence of these dimensions on the resulting hydrophobic characteristics of the surface [67,68];

- Plasma treatment can be used both for surface etching and for the generation of nanoscale roughness. Surface morphology is evaluated using the root mean square roughness (RMS) and the R_a_ parameter. The effect of plasma treatment is nonlinear: at low discharge power, the surface may become smoother and even hydrophilic, whereas at high power, roughness favorable for hydrophobicity is generated. In addition, plasma treatment can modify the surface chemical composition (for example, by introducing oxygen-containing functional groups), which also affects wettability [69,70];

- Hydrothermal synthesis promotes the formation of various types of nanostructures on the substrate (nanofibers, nanorods, flower-like, or pine tree-like morphologies), providing the required scale of roughness. The key parameters are the size and density of the nanostructures, which can be controlled by varying the concentration of the reagents (e.g., NaOH). The hierarchical nature of the structure also has a significant influence on hydrophobic properties, since the combination of features of different scales enables a more pronounced effect through more efficient retention of the trapped air layer [5,71].

#### 2.1.1. Etching

Etching is an effective approach for fabricating hydrophobic and superhydrophobic coatings by creating hierarchical surface roughness on the substrate followed by a reduction in its surface energy. The concept is inspired by natural analogues, such as lotus leaves, where the combination of micro- and nanoscale surface relief with low surface energy provides pronounced water repellency. The method generally involves a two-step approach. First, the required micro- and nanoscale surface relief, consisting of protrusions, pits, and pores, is generated by selectively exposing the substrate to a chemical etchant (acids, alkalis, or their mixtures). The key process parameters include the solution concentration, etching time, and process temperature, while the use of protective masks enables the fabrication of more complex and localized patterns. In the second step, the etched surface is modified with hydrophobizing agents, such as fluorinated silanes (including hexadecyltrimethoxysilane and PFTS) or carboxylic acids (stearic acid and myristic acid), whose molecules are adsorbed onto the rough surface, further reducing its surface energy. As a result, water forms droplets on the surface that interact only weakly with the substrate.

The method is applicable to a wide range of materials; however, it is most commonly employed for metals and alloys (aluminum and its alloys (e.g., the Russian grades AD31 and AMg6), as well as copper, magnesium, and stainless steel) and a number of non-metallic substrates, including glass and certain polymers (e.g., PET track-etched membranes). The major advantages of this approach include the possibility of precise control over the resulting surface structure by adjusting the etching parameters, the high degree of coating integration with the substrate (since the surface relief is generated directly within the substrate material rather than being deposited as a separate layer), and its relative simplicity and scalability, making the method suitable for industrial implementation. In addition to imparting hydrophobic or superhydrophobic properties, such coatings often exhibit other beneficial effects, including self-cleaning capability, improved corrosion resistance, and reduced ice adhesion [72,73,74].

**Figure 2:**
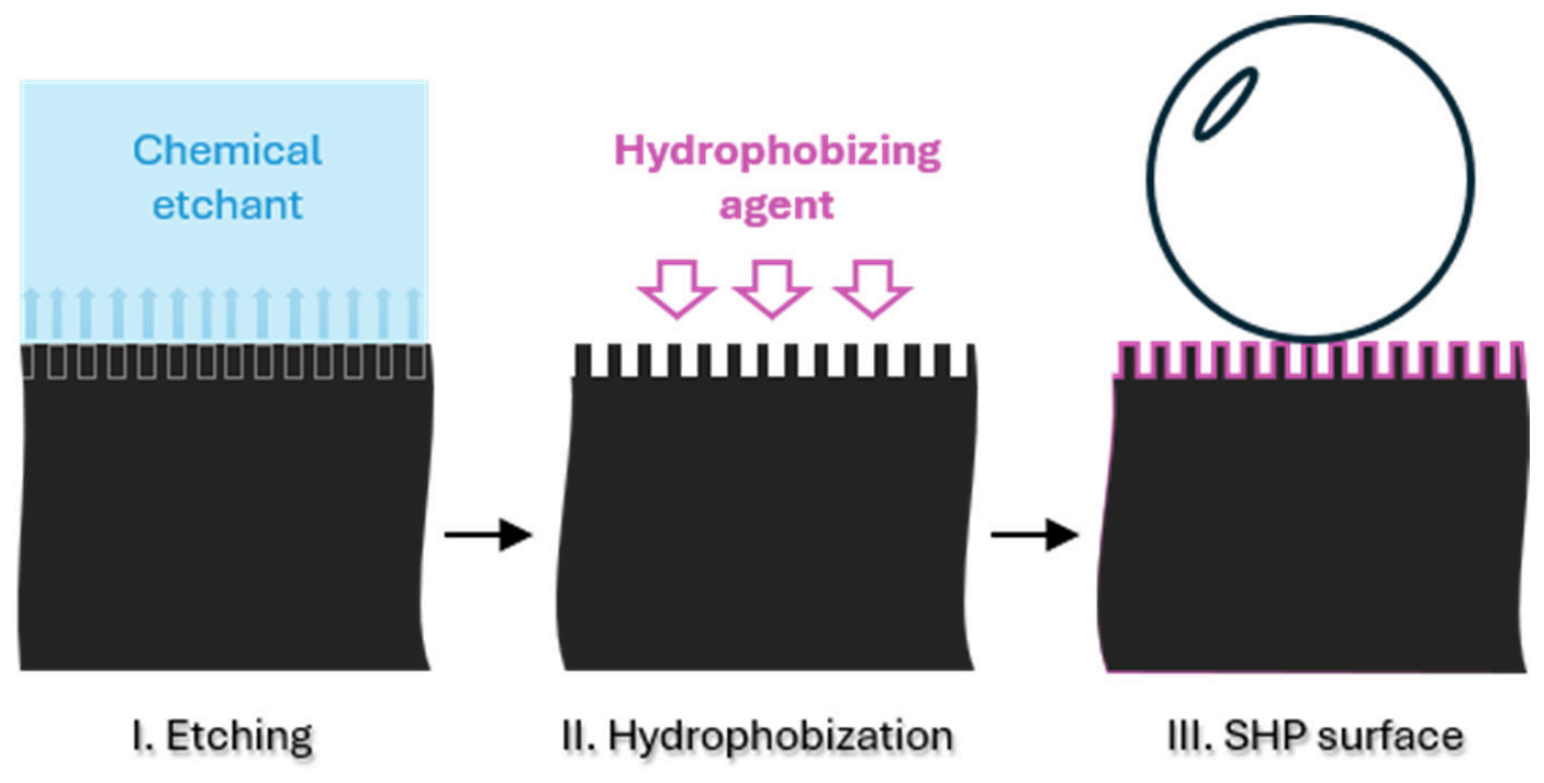
The etching process diagram. Blue arrows indicate the partial dissolution of the surface material during etching. Pink arrows indicate the application of the hydrophobizing agent.

Nevertheless, the method is associated with several limitations and potential drawbacks. The use of aggressive chemical reagents requires strict compliance with safety regulations and careful consideration of waste disposal. Excessive etching caused by exceeding optimal exposure time may damage the surface structure or deteriorate the mechanical properties of the substrate. The highly developed rough morphology responsible for the functional properties of the coating is often susceptible to mechanical damage: friction, impact, or abrasive wear may destroy the surface microfeatures, resulting in the loss of the superhydrophobic effect. Furthermore, in certain service environments (e.g., under extreme pH conditions), degradation of both the surface modification layer and the structured surface itself may occur. Finally, the effectiveness of the method strongly depends on the nature of the material being treated, since not all substrates are equally amenable to chemical etching, and each requires an individually selected etchant [75,76,77]. The schematic illustration of the etching process is shown in Figure 2.

#### 2.1.2. Anodization

Anodization is widely employed as the fundamental step in the fabrication of hydrophobic and superhydrophobic coatings; however, it is important to recognize that the porous oxide film formed during this process is inherently hydrophilic, and the desired water-repellent effect is achieved only through subsequent surface modification. The principle of the method is based on creating a well-developed micro- and nanoscale surface relief (hierarchical roughness) on a metallic substrate by anodization, followed by a reduction in the surface energy of the resulting structure through treatment with a hydrophobizing agent. The emergence of a hydrophobic or superhydrophobic state is governed by the interaction between a water droplet and the rough surface: in the presence of multiscale surface features, the droplet partially traps air within the surface depressions, thereby minimizing its contact with the solid substrate. For the development of superhydrophobicity, characterized by a contact angle exceeding 150°, multimodal roughness plays the decisive role [78,79].

**Figure 3:**
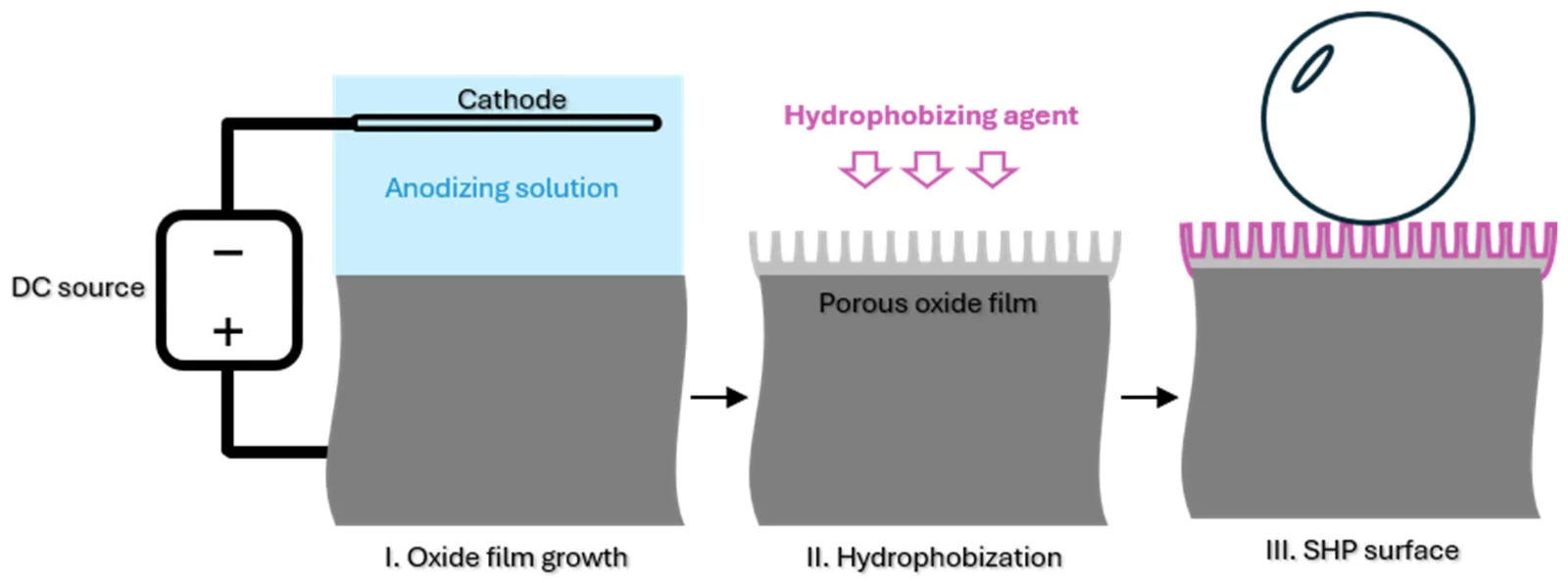
Generalized scheme of anodizing followed by hydrophobization.

The technological process is generally carried out in two stages. During the first stage (anodization), the workpiece is immersed in an electrolyte and connected as the anode to a direct current power supply. The morphology of the resulting oxide layer varies over a wide range, from relatively dense structures to highly porous and hierarchical structures, and is determined by a combination of parameters, including the type of metal, electrolyte composition, voltage, current density, and temperature. For example, mixtures of sulfuric and phosphoric acids are commonly used for the anodization of aluminum, whereas fluoride-containing electrolytes are employed for tantalum and niobium. The second stage (hydrophobization) involves the application of compounds that reduce the surface energy of the porous oxide layer through adsorption (Figure 3). Fluoroalkylsilanes, stearic acid, octadecyltrimethoxysilane, selected copolymers, and, in some cases, combined formulations (e.g., a mixture of stearic acid and dimethyl sulfoxide) are used as hydrophobizing agents. As a result, a low-surface-energy layer is formed on the structured surface, providing the required water-repellent properties [80,81,82,83].

The most common application of this method is the treatment of aluminum and its alloys; however, anodization has also been successfully applied to tantalum, niobium, and titanium. At the same time, each metal requires the individual selection of the electrolyte and processing conditions to achieve the optimal oxide structure. The anodization of stainless steel is associated with additional challenges: owing to the presence of a naturally formed passive oxide layer, the process requires special conditions, and the resulting coatings often exhibit lower stability [84,85,86].

The advantages of the method include the possibility of precisely controlling the morphology of the oxide layer, including the pore size, pore distribution, and the formation of hierarchical structures that directly influence the degree of hydrophobicity. The porous oxide film serves as an effective substrate for the subsequent deposition of other coatings (e.g., polymers and composites), providing enhanced adhesion compared with a smooth metallic surface. Furthermore, the anodic oxide layer itself contributes to improved corrosion resistance and wear resistance of the substrate. At the same time, the method is highly sensitive to deviations in the processing parameters. Even minor changes in the electrolyte composition, temperature, applied voltage, or treatment duration can substantially affect the resulting structure. Additional limitations include the potential long-term instability of the hydrophobic layer (due to the leaching or degradation of the hydrophobizing agent under the action of moisture, aggressive media, or mechanical loading), as well as the susceptibility of superhydrophobic coatings to abrasion, scratching, and other forms of mechanical damage. For certain materials (particularly stainless steel), the implementation of the method is associated with greater technological complexity and less predictable results [80,81,82,83,84,85,86].

#### 2.1.3. Laser Treatment

**Figure 4:**
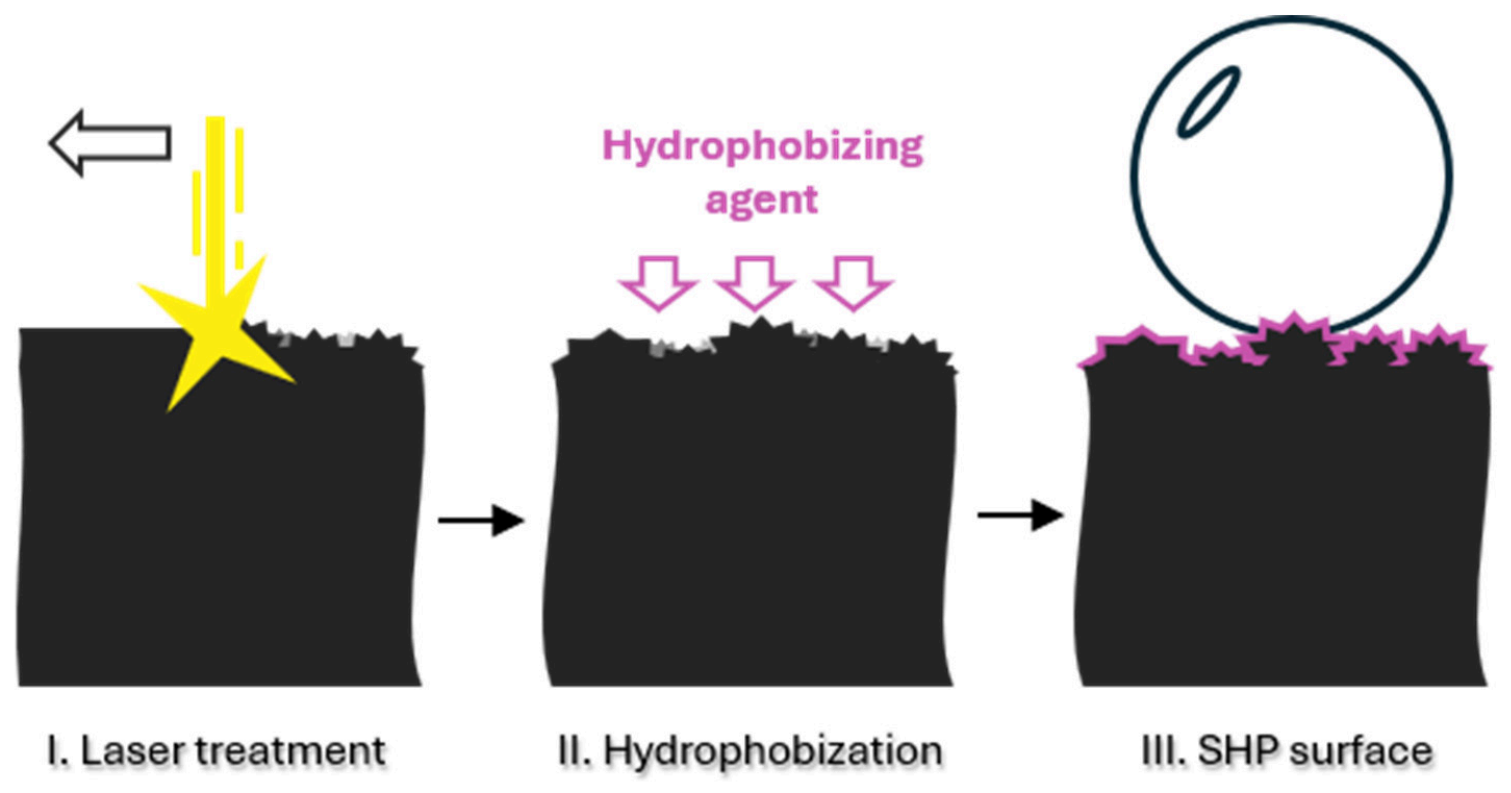
Generalized scheme of laser treatment. Black-and-white arrow indicates the movement of the laser beam across the surface. Pink arrows indicate the application of the hydrophobizing agent. The four-pointed yellow star indicates the laser irradiation spot on the surface.

Laser treatment is a method for creating hydrophobic and superhydrophobic coatings through controlled modification of surface topography using laser irradiation. Laser–material interaction leads to localized heating, melting, and partial ablation of the material, resulting in the formation of surface relief structures such as pits, grooves, grids, or cone-shaped and hierarchical textures (Figure 4). This alters surface wettability and induces water-repellent properties. Hydrophobic and superhydrophobic states are achieved through two key mechanisms: (1) micro- and nanostructuring of the surface, which creates composite solid–liquid–gas interfaces and enables Wenzel or Cassie–Baxter wetting regimes depending on geometry; (2) reduction in surface energy via chemical modification. Surface energy reduction is achieved using agents such as fluoropolymers, perfluorinated compounds, organosilicon compounds (organosilanes), copolymers of glycidyl methacrylate and fluoroalkyl methacrylates, modified nanoparticles (SiO_2_), stearic acid, phosphoric acids, and polydimethylsiloxane (PDMS). Laser texturing is applicable to metals and alloys (stainless steel, copper, titanium, magnesium, tungsten, aluminum alloys, etc.), ceramics (Si_3_N_4_, Al_2_O_3_, ZrO_2_), as well as polymeric and composite materials [87,88,89].

**Figure 5:**
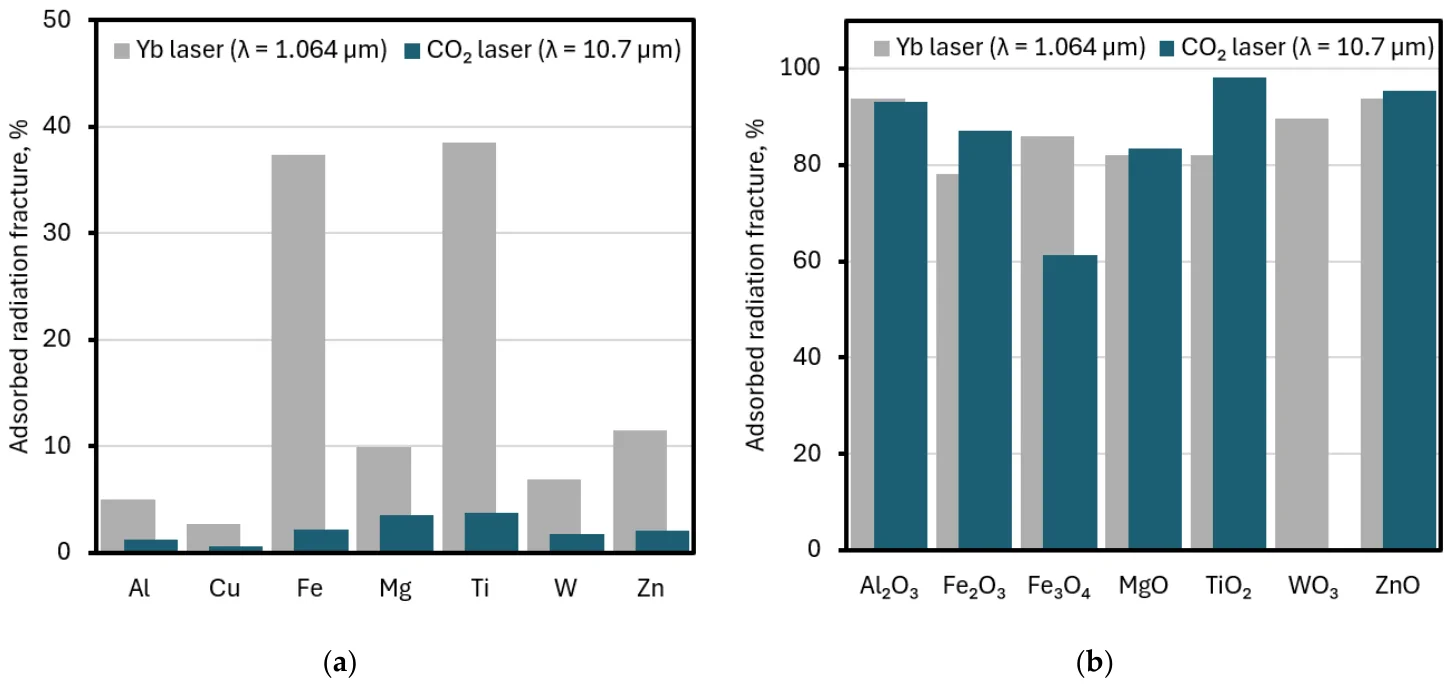
Absorbance of infrared radiation from Yb and CO_2_ lasers by various materials: (**a**) metals; (**b**) corresponding oxides.

Key parameters for hydrophobic and superhydrophobic surface formation include laser wavelength and the spacing between surface features, which determine micro- and nanostructure formation and thus wettability. Infrared lasers, such as ytterbium fiber lasers with a wavelength of 1.064 µm, are commonly used due to efficient absorption by metals, enabling surface structuring through localized heating, melting, and ablation (Figure 5) [22,23,24].

Thus, metal oxides exhibit a high degree of infrared (IR) absorption, which, on the one hand, can increase the efficiency of laser processing but on the other hand leads to the removal of a significant portion of surface oxides due to intensive ablation. This is related to the fact that laser power is selected based on the need to texture the metal surface rather than the oxide layer, which requires an increase in irradiation power. As for the scanning step, its values should not exceed 100 µm. The choice of laser type is determined by the material and the required surface relief characteristics: femtosecond lasers provide the highest precision (±0.1 µm) and minimal thermal impact on the material and are suitable for virtually all materials, including ceramics and polymers; picosecond lasers represent a compromise between processing quality and productivity and are commonly used for metal processing; nanosecond lasers are also widely applied for surface texturing, particularly for metallic materials [90,91,92].

Despite significant progress in modelling laser–material interaction, laser processing parameters are most often determined experimentally, since their theoretical calculation requires consideration of a large number of interdependent factors. These include laser power, material absorption coefficient, scanning speed and hatch spacing, pulse repetition rate, focused beam diameter, as well as thermophysical properties of the material (density, heat capacity, thermal conductivity, phase transition temperatures and latent heats) [92]. Additional effects arise from the initial surface state and morphology, which influence energy absorption and redistribution. The combination of these factors makes it difficult to derive a universal analytical expression for optimal processing conditions.

The advantages of this technology include high precision, controlled depth and geometry of surface structures, non-contact and localized processing, environmental friendliness, and the ability to create multifunctional surfaces. The disadvantages include limited processing speed, risk of thermal damage to the material, difficulty in achieving “ideal” superhydrophobicity without additional chemical modification, low wear resistance of the formed structures, and strong sensitivity to surface cleanliness [22,23,24,90,91,92].

#### 2.1.4. Lithography

**Figure 6:**
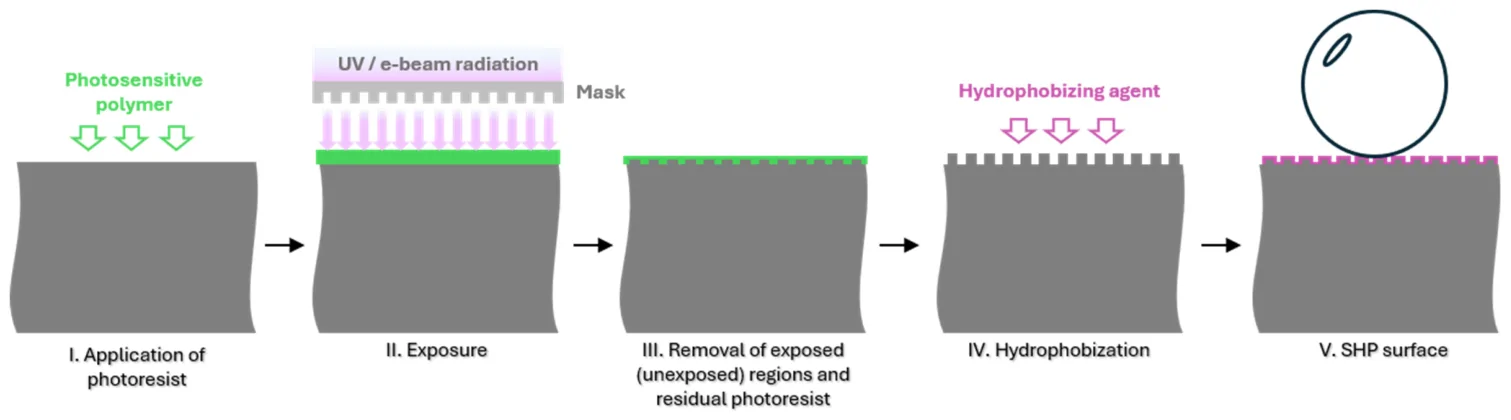
Generalized scheme for obtaining HP and SHP coatings by lithography. The green, purple, and pink arrows indicate the application of the photosensitive polymer, UV/e-beam irradiation, and hydrophobizing agent, respectively.

Lithography is a technology for fabricating micro- and nanostructures on a substrate surface using masks and radiation sources (UV, X-ray, electron beam). The process of forming hydrophobic (HP) and superhydrophobic (SHP) coatings includes several stages. First, the substrate is cleaned and activated—degreased and treated by plasma or etching to improve adhesion. Then a photosensitive polymer layer (photoresist, either positive or negative type) is applied. This is followed by exposure through a mask using UV light or an electron beam to define the pattern. During the development step, either the exposed or unexposed regions of the photoresist are removed depending on its type, forming a patterned structure (Figure 6). The resulting relief is then transferred to the substrate via etching or filled with material through deposition processes. After that, the surface is hydrophobized using fluorinated compounds (e.g., fluorooctyltrichlorosilane), organosilicon polymers, or alkylsilanes. If necessary, residual photoresist is removed, leaving a structured hydrophobic surface. The process is completed by final treatment steps such as rinsing, drying, and, if required, thermal stabilization of the coating [93,94,95].

HP and SHP coatings fabricated via lithography can be applied to a variety of substrates, including metals (gold, aluminum and their alloys), silicon, polymers (polymethyl methacrylate, polydimethylsiloxane), glass with conductive layers, and ceramics, provided they are compatible with processing conditions. This method offers several advantages, namely very high resolution (10–100 nm) and precise control over geometry and positioning of features, enabling complex hierarchical patterns with defined topology. It is highly reproducible, with minimal variation between fabricated structures. The method is compatible with microelectronics and can be readily integrated into existing technological lines. In addition, lithography allows the fabrication of a wide variety of patterns, from regular arrays of pillars to arbitrary designs, and is applicable to both micro- and nanostructures [95,96,97,98].

However, its practical implementation faces several limitations. High-cost equipment (UV exposure systems, electron-beam lithography tools, positioning systems) and multistep processing make the technology expensive. Limited processing area per cycle complicates scaling to large surfaces without stitching artifacts. Even minor mask defects (dust, scratches, irregularities) can lead to defective coatings. The high cost of fluorinated reagents further increases production expenses. The method is poorly suited for mass production due to low scalability and high labor intensity. In addition, not all materials can withstand harsh processing conditions (high temperatures, aggressive etchants), and the resulting fine micro- and nanostructures may degrade under mechanical stress or service conditions [95,98].

#### 2.1.5. Plasma Treatment

**Figure 7:**
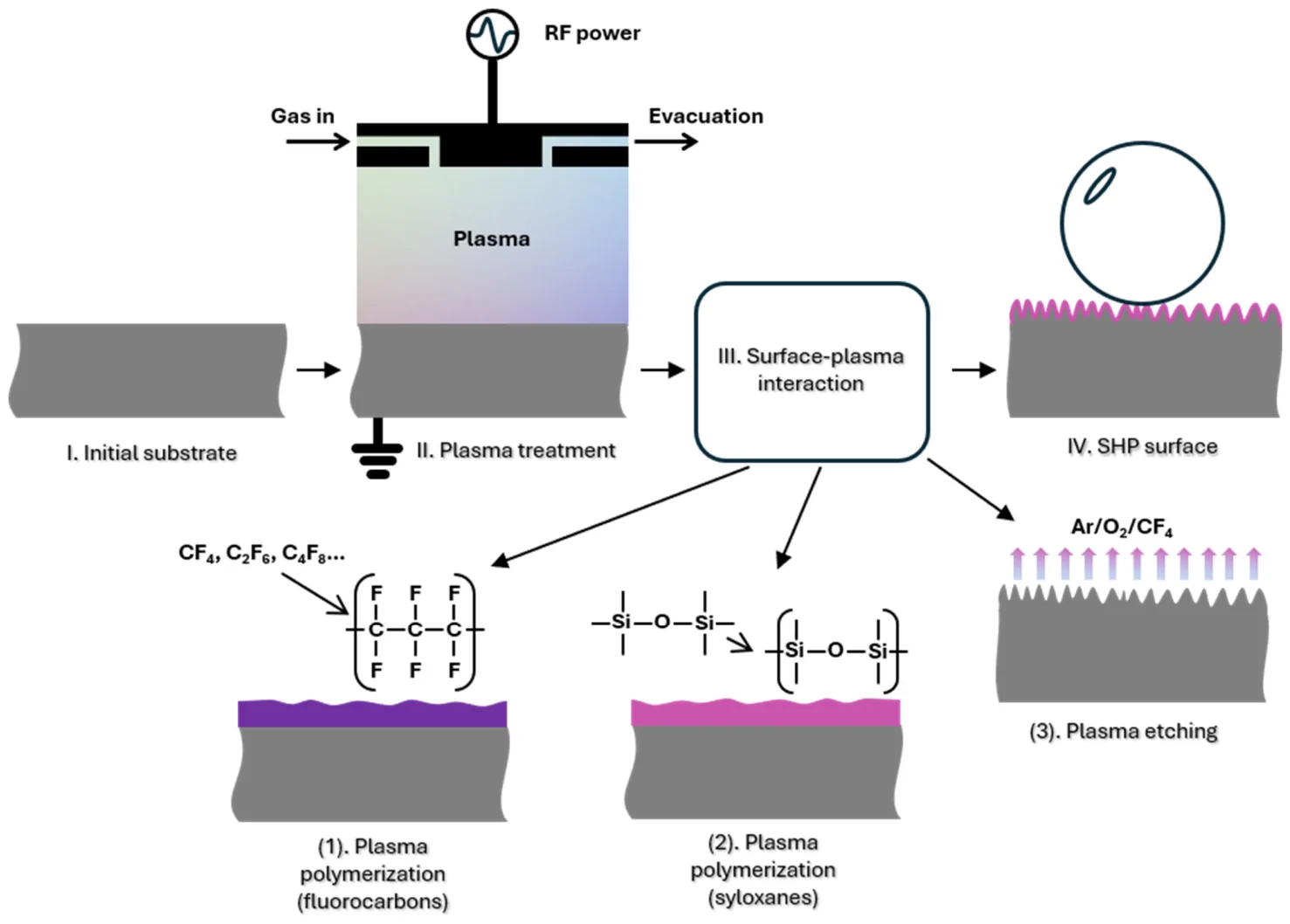
Generalized scheme of plasma treatment. The colored gradient represents the plasma, while the arrows indicate the partial removal of surface material due to its interaction with the plasma.

Plasma treatment is a surface modification method using highly reactive plasma species such as electrons, ions, and radicals. Interaction of these species with the substrate alters its chemical structure and surface topology. Hydrophobic and superhydrophobic coatings can be obtained under several conditions: (1) Fluorocarbon plasmas (CF_4_, C_2_F_6_, C_4_F_8_ and similar gases) induce plasma polymerization, leading to deposition of fluorocarbon layers structurally similar to polytetrafluoroethylene, providing low surface energy and strong hydrophobicity; this process can be carried out under both atmospheric and vacuum conditions. (2) Plasma polymerization using organic monomers (e.g., hexamethyldisiloxane) results in polymer-like coatings with hydrophobic properties, formed directly within the plasma chamber without additional processing steps. (3) Plasma etching can create micro- and nanostructured roughness, enhancing intrinsic hydrophobicity to superhydrophobic levels, particularly in polymers and certain metals, where combined texture and low surface energy result in contact angles exceeding 150° (Figure 7) [99,100,101].

Advantages of plasma technology include process simplification (shorter production time, reduced equipment requirements, elimination of additional processing steps), environmental benefits (no liquid waste from washing or cleaning), cost efficiency, applicability to a wide range of materials (polymers, metals, ceramics, composites), ability to process complex shapes, and strong adhesion of coatings to substrates. Disadvantages include poor long-term durability of superhydrophobic coatings on metals (initial contact angles > 150° decrease rapidly due to oxidation, contamination of micro- and nanostructures, and mechanical wear), requiring additional chemical modification for practical applications; instability over time; difficulty in controlling surface roughness; high energy consumption for plasma generation; potential damage to thermally sensitive materials; and strong sensitivity to process parameters (pressure, discharge power, exposure time, and gas composition) [102,103,104,105].

#### 2.1.6. Hydrothermal Synthesis

**Figure 8:**
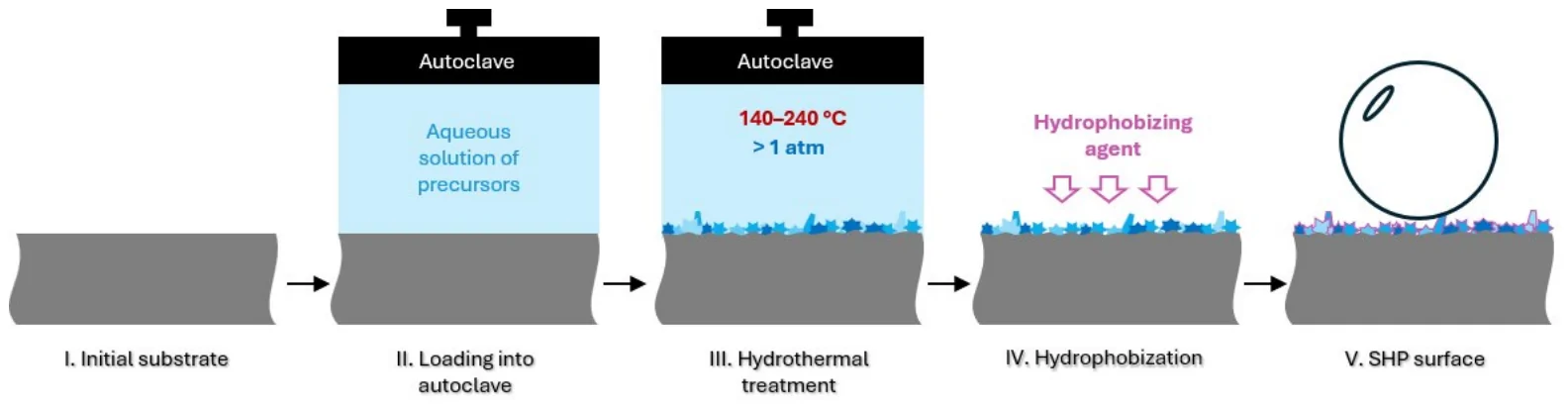
Generalized scheme of hydrothermal synthesis. The different shades of blue represent crystals formed during hydrothermal synthesis, with the different shades used only for visual distinction. The pink contour represents the hydrophobizing agent deposited on the surface.

Hydrothermal synthesis is a method for producing materials through chemical reactions in a sealed vessel (autoclave) at temperatures above 100 °C and pressures above 1 atm. A solution containing precursors (metal salts, metal hydroxides, acids, etc.) is placed into the autoclave and heated to typically 140–240 °C or higher for 2–48 h or longer. Under these conditions, precursors dissolve and subsequently crystallize either in the bulk solution or on seed crystals, forming nanostructures such as nanoparticles or nanorods. These structures can generate surface roughness, which combined with material chemistry may provide hydrophobic or superhydrophobic properties, although additional surface modification is often required. After treatment, samples are typically rinsed with distilled water, dried at ~100 °C for several hours, and sometimes subjected to additional heat treatment (e.g., 400 °C) to stabilize crystalline structure (Figure 8) [106,107,108].

This technique can be applied to various substrates, including metals and alloys (e.g., magnesium and aluminum alloys), metal oxides (Al_2_O_3_), ceramics, glass, and some polymers resistant to high temperatures and pressures. The main requirement is chemical stability under hydrothermal conditions and compatibility with the growing structures. Advantages include controllable morphology and particle size through adjustment of synthesis parameters (temperature, pressure, precursor concentration, time), enabling tuning of surface structure and wettability, as well as good structural ordering of the resulting coatings. Disadvantages include long processing times, need for expensive high-pressure autoclaves, difficulties in scaling up, and inability to directly observe the process inside the reactor [109,110,111,112].

### 2.2. Methods of Applying Functional Layers for the Formation of HP and SHP Coatings

Methods in this group enable the formation of coatings as either continuous or structured layers, while in some cases the surface relief develops directly during the deposition process or through structural self-organization. The key advantage of these approaches is the possibility of combining surface morphology formation and the incorporation of hydrophobic components within a single technological process, which is particularly valuable for scalable solutions. The main methods belonging to this group are presented below:

- Sol–gel allows the fabrication of porous layers with controlled morphology and is widely used for the incorporation of hydrophobic precursors (e.g., alkoxysilanes) already at the sol stage. The important quantitative characteristics include porosity and pore size distribution (e.g., pores ranging from 200 to 1300 nm when phenyltrimethoxysilane is used); the coating thickness is typically tens to hundreds of nanometers and may reach several micrometers after multiple deposition cycles; roughness (R_a_, RMS) depends on the sol composition, drying rate, and heat-treatment conditions; with appropriate surface modification (e.g., using PTMS), the contact angle can reach 130–160° [113,114,115].

- Wet chemical reactions in solution enable the deposition of functional layers through controlled reactions occurring on the substrate surface. The principal parameters include coating uniformity and thickness, which depend on the reagent concentration, temperature, and treatment duration; the morphology of the deposited layer (grain size and the presence of nano- and microstructures) is typically evaluated by scanning electron microscopy and profilometry; the contact angle frequently reaches 140–155° when suitable modifiers are employed (e.g., by adjusting the pH and adding surfactants) [116,117];

- Dip coating—immersion of the substrate into a solution followed by withdrawal—enables the formation of thin coatings, while multiple deposition cycles allow precise control of the coating thickness and structure. The key characteristics include the coating thickness per deposition cycle, which is typically tens to hundreds of nanometers and depends on the withdrawal rate and solution viscosity; coating uniformity is commonly evaluated using profilometric measurements and optical microscopy; roughness and porosity can be controlled by the solution composition and drying conditions; the contact angle is an important parameter for evaluating the functional properties and generally exceeds 150° [5,118];

- Spray coating, involving the rapid spraying of a suspension or solution, is well suited for scaling up and for the treatment of large-sized objects. The main quantitative parameters include coating thickness and uniformity, which depend on the spraying pressure, stand-off distance, droplet size, and solution viscosity; coating density and porosity are determined by the spraying conditions and suspension composition; the mechanical durability is frequently evaluated by abrasion testing (e.g., 50 abrasion cycles) or the tape test [119,120];

- Electrodeposition provides the possibility of controlling the coating morphology through the applied current parameters and electrolyte composition. The important characteristics include the thickness of the deposited layer, which is controlled by the electric charge passed through the electrochemical cell and the current density; the surface morphology (porosity, grain structure, and columnar or globular morphology) depends on the applied potential, electrolyte composition, and additives; the surface roughness (Ra, Sa) can be deliberately increased to enhance hydrophobicity; adhesion to the substrate is evaluated by scratch testing or pull-off testing; the contact angle reaches 150° or higher when hydrophobic additives are incorporated or when subsequent surface modification is applied; in several studies, values as high as 160° have been reported for hierarchically structured coatings produced under pulsed deposition conditions [121,122,123];

- Thermal spraying enables the deposition of dense or porous coatings with high adhesion. The surface relief is formed as a result of particle impact dynamics and spraying conditions. The principal process parameters include the coating thickness, which may range from tens to hundreds of micrometers; the porosity and pore size depend on the particle temperature and velocity, powder composition, and spray distance; the surface roughness (R_a_, S_z_) is determined by the particle size and shape, as well as the spray jet parameters; the adhesion strength is evaluated by pull-off testing or scratch testing; the contact angle of superhydrophobic coatings may exceed 150° when hydrophobic components are incorporated into the coating or when additional surface modification is performed (e.g., by combining thermal spraying with laser treatment) [124,125].

#### 2.2.1. Sol–Gel Method

Sol–gel technology is a method for producing coatings through the transformation of a liquid solution into a gel, followed by its conversion into a solid coating. Initially, hydrolysis of the precursor takes place, followed by subsequent polycondensation reactions leading to the formation of a sol. The sol then gradually transforms into a gel, which is subsequently applied onto the desired substrate. Finally, the coating is dried and subjected to thermal treatment. The sol can be deposited onto the substrate using several methods, including dip coating, spin coating, and spraying. The choice of deposition method depends on the shape and size of the substrate. This technology is widely used for the fabrication of hydrophobic and superhydrophobic coatings on various substrates, including metals, glass, ceramics, wood, and other materials. To achieve hydrophobic and superhydrophobic properties, hydrophobizing agents are used as precursors, such as fluorinated compounds (fluorosilanes, fluoropolymers), organosilicon compounds (trimethoxymethylsilane, dimethyldichlorosilane), or hydrophobized fillers (aerosil). In the fabrication of superhydrophobic coatings, a critically important step is the preliminary preparation of the substrate: prior to the deposition of the functional layer, the substrate surface is textured to create the required micro- or nanoscale roughness, which ensures the desired water-repellent properties [126,127,128].

**Figure 9:**
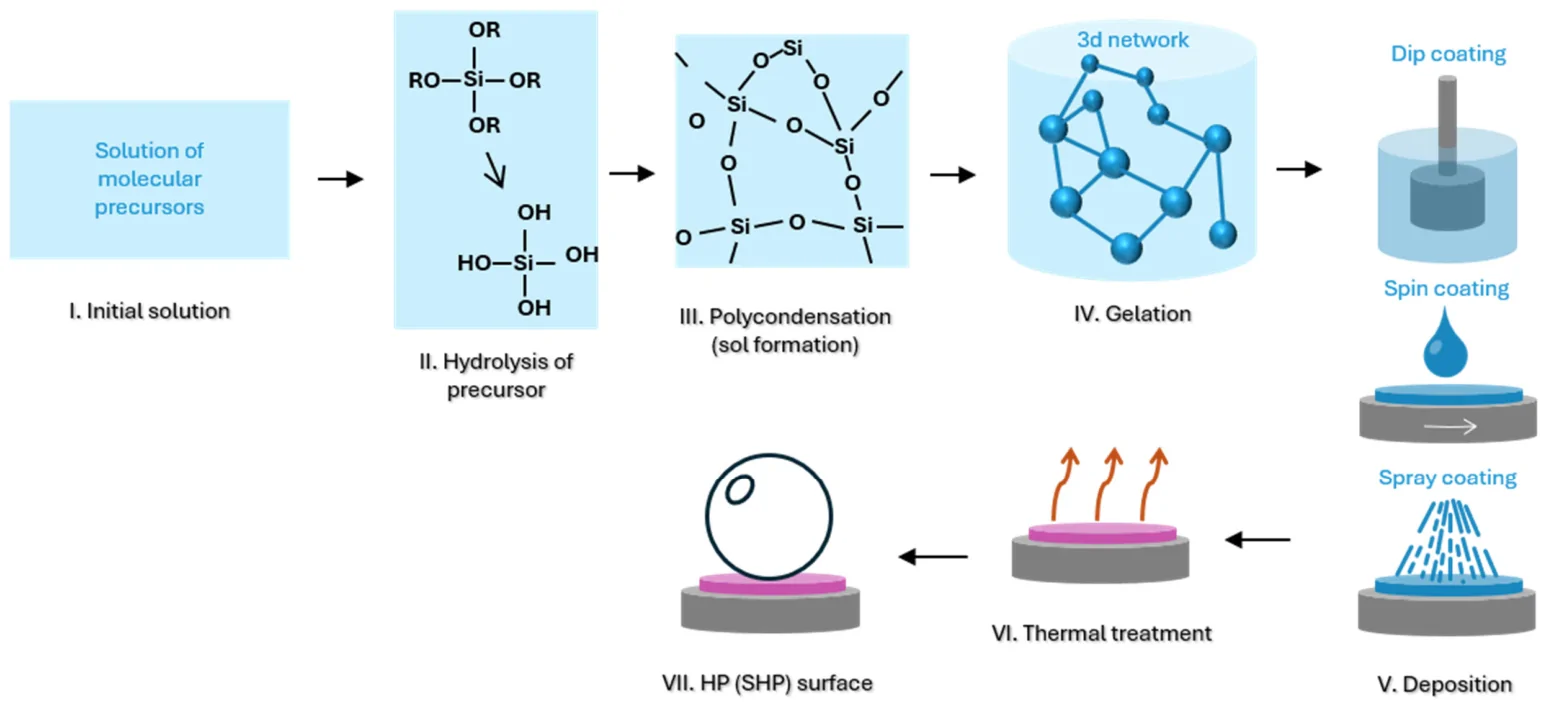
Generalized scheme of sol–gel technology for HP and SHP coatings. Arrows indicate the process of heating.

Sol–gel technology demonstrates several significant advantages, including the possibility of coating a wide range of substrates, precise control over the structure and composition of the formed layer, and high thermal stability. However, its limitations should also be considered, such as the achievable coating thickness, the risk of crack formation, the potential negative impact of thermal treatment on substrate integrity, and relatively low mechanical durability [129,130,131,132]. A generalized scheme for the formation of HP and SHP coatings using the sol–gel method is shown in Figure 9.

#### 2.2.2. Wet Chemical Reaction

Wet chemical reaction is a method for producing hydrophobic and superhydrophobic coatings involving surface treatment with solutions containing hydrophobic or functional components, followed by formation of a protective film or structured layer. This method is relatively simple, allows processing of substrates of various sizes and shapes, and enables control over morphology and particle size. Wet chemical approaches include sol–gel processes, wet etching, and immersion-based techniques. Hydrophobizing agents may be introduced during synthesis or applied as post-treatment. Common materials used include organosilanes (vinyltrimethoxysilane, triethoxysilane, (tridecafluoro-1,1,2,2-tetrahydrooctyl)dimethylchlorosilane), fluorinated compounds (fluoropolymers, perfluorinated compounds, fluorourethane emulsions), modified nanoparticles (silica, alumina nanoparticles modified with stearic acid, calcium carbonate), and polymer matrices (polyacrylates, polyurethane systems modified with silicate components). Acids and other reagents (e.g., citric acid combined with tetraethyl orthosilicate in sol–gel systems) are also used [52,133,134].

Advantages include versatility, control over morphology and particle size, relative simplicity and accessibility, ability to form hierarchical structures, and cost-effectiveness compared with methods such as electrodeposition, CVD, and plasma treatment. Disadvantages include limitations in crack resistance and coating thickness, risk of substrate degradation during thermal treatment, low mechanical durability of superhydrophobic coatings, need for careful surface preparation to ensure adhesion, and possibility of defect formation due to non-uniform deposition or solvent evaporation [52,135,136,137].

#### 2.2.3. The Method of Dip Coating

Dip coating is a coating deposition technique in which substrates are fully immersed in a solution followed by controlled withdrawal. Hydrophobicity or superhydrophobicity is achieved through a combination of hierarchical surface roughness (micro- and nanostructures) and low surface energy provided by hydrophobizing agents. Substrates used for HP and SHP coatings fabricated via the dip coating method include metals, polymers, glass, ceramics, textiles, paper, composites, and porous materials. Prior to coating deposition, surface preparation is required, including cleaning, degreasing, and drying, and, if necessary, surface activation (plasma treatment or etching). The substrate is then immersed in a modifying solution for a specified time. The coating solution may include components for different functions: nanoparticles (SiO_2_, Al_2_O_3_, carbon nanotubes, etc.) to create surface roughness; polymers as the coating matrix (polytetrafluoroethylene, polyurethanes, acrylic polymers); hydrophobizing agents to reduce surface energy (fluorosilanes, organosiloxanes, stearic acid); and solvents as dispersion media (ethanol, hexane, water, etc.). Subsequently, the coated substrate is withdrawn at a controlled speed. The thickness of the liquid layer on the substrate is determined by the withdrawal speed (ranging from mm/s to cm/s) and also depends on solution viscosity, liquid density, and surface tension. Finally, film formation occurs through solvent evaporation, nanoparticle and polymer deposition on the surface, and curing at room temperature or under heating [138,139,140].

The advantages of this technique include precise control over coating thickness via adjustment of withdrawal speed and solution viscosity, uniform deposition of homogeneous layers even on complex geometries and large-area substrates, as well as simplicity and cost-effectiveness, making the process suitable for industrial-scale production. The disadvantages of the dip coating method include limitations in substrate compatibility (high adhesion is not always achieved), strong dependence of coating quality and thickness on process parameters (solution viscosity, withdrawal speed, temperature), and the risk of defect formation (air bubbles, trapped solvent). In addition, the method is limited in scalability for large-scale objects and mass production. To achieve superhydrophobic properties, additional post-treatment (hydrophobization or thermal treatment) is often required, and the resulting coatings typically exhibit low mechanical durability and sensitivity to damage [141,142,143].

#### 2.2.4. Spray Coating Technology

Spray coating is a technique for depositing coatings onto various surfaces by spraying a suspension or solution of a modifying agent, enabling the formation of HP and SHP coatings. During spraying, the material in the form of a solution, emulsion, or suspension is delivered onto the substrate through a spray nozzle under controlled pressure. The particles are uniformly distributed over the surface, forming a layer of controlled thickness. After deposition, solvent evaporation, polymerization, curing, or other processes occur, resulting in the formation of a stable coating. The spray method enables coating of a wide range of materials, including metals, glass, ceramics, concrete, cellulose, textiles, plastics, and wood. The process is efficient for industrial-scale production and large-area processing and is suitable for substrates of different nature and geometry. Nanoparticles, fluorinated compounds, silicones, and other components can be incorporated into the coating to improve hydrophobic properties, mechanical strength, and durability [144,145].

Compared with some other technologies (electrodeposition, CVD, laser texturing, lithography, etc.), this method does not require complex equipment and is relatively simple to implement. However, it also has several disadvantages: it is difficult to achieve strictly controlled surface roughness, which affects the WCA; coatings often exhibit insufficient wear resistance and degrade rapidly under friction or mechanical stress; some materials (e.g., fluoropolymers) show poor adhesion to substrates and require additional adhesion promoters or surface pretreatment; the results strongly depend on process parameters such as spray distance, pressure, deposition rate, and other conditions; furthermore, structural defects (inhomogeneities, pores) may form, reducing hydrophobic and superhydrophobic performance [146,147,148].

#### 2.2.5. Electrodeposition

Electrodeposition is a method for forming coatings on conductive substrates under the action of electric current, where film-forming ions are reduced at the cathode (substrate), resulting in coating formation. Process parameters (current density, time, electrolyte composition) influence the structure and properties of the coating. The choice of electrodeposition method (pulsed/impulse electrodeposition, anodization, co-deposition of metals and organic ligands, electrophoretic deposition, electrodeposition from ionic liquids, plasma electrolytic oxidation, etc.) for producing HP and SHP coatings depends on the required properties (contact angle, wear resistance, electrical conductivity), substrate type, and economic factors. To achieve hydrophobic and superhydrophobic properties, it is necessary to create a rough surface (micro- and nanostructures) and reduce surface energy using hydrophobic additives or subsequent surface treatment. Texturing during electrodeposition can be achieved by controlling process parameters. Under diffusion-controlled conditions, dendritic structures may form, creating surface roughness. Variation in current density and deposition time can also increase surface roughness. The introduction of nanoscale particles or combinations of metals and nonmetals into the electrolyte leads to co-deposition of nanoparticle agglomerates, resulting in submicro- and microstructures and multimodal roughness, which is particularly important for composite electrochemical coatings. Similar effects are observed during the deposition of composites such as Cu–MoS_2_ or Cr–Nb_2_N + Ta_2_N. Hydrophobic additives used in electrodeposition of HP and SHP coatings include carboxylic acids and their derivatives, fluorinated organic compounds (including fluoropolymers and fluoroorganosilanes), and nanoparticles (SiO_2_, ZnO, Al_2_O_3_, graphene, etc.) [149,150,151].

**Figure 10:**
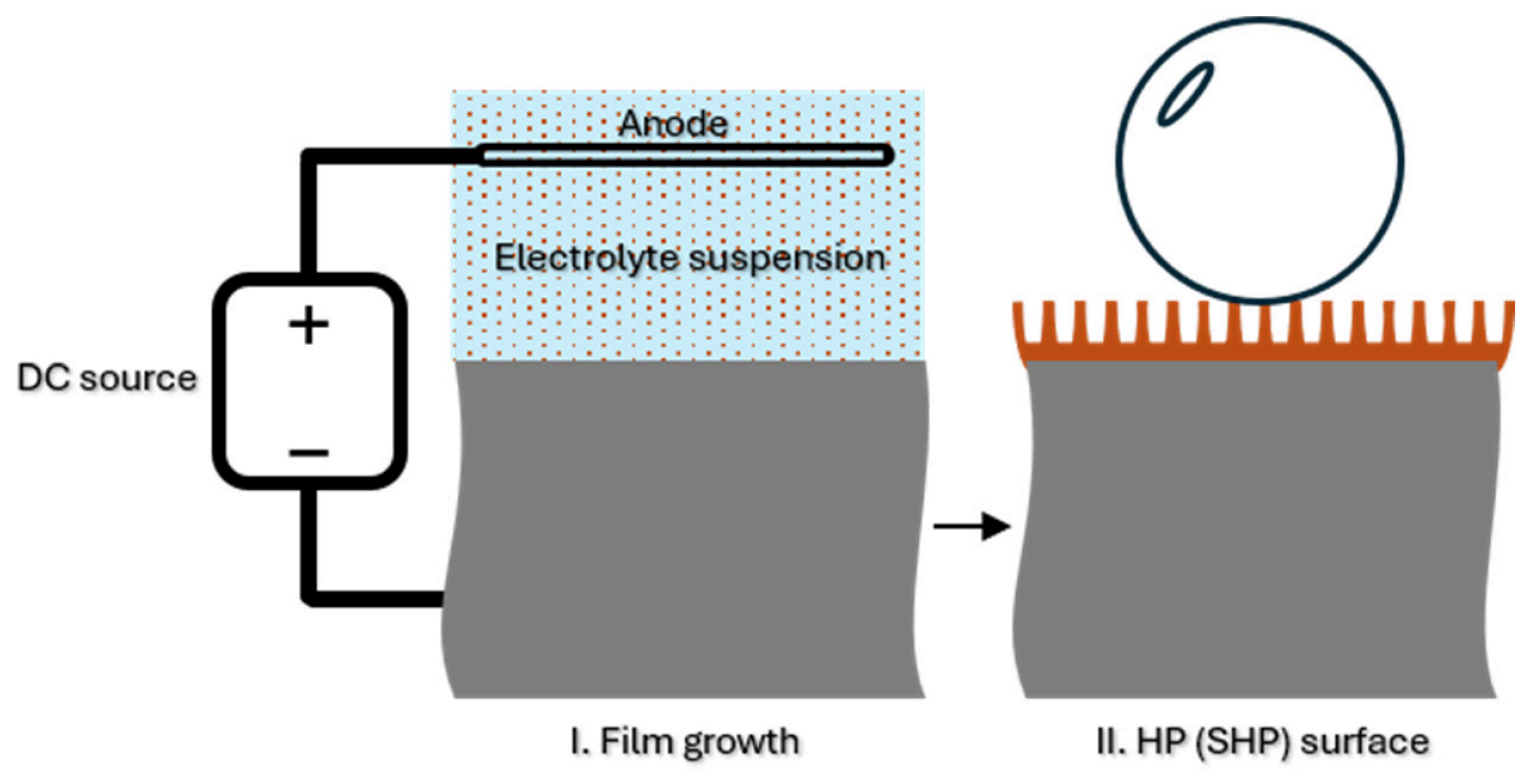
Generalized scheme of electrodeposition for HP and SHP coatings.

The advantages of electrodeposition include technological simplicity and scalability, low energy consumption, and precise control of process parameters. The method enables coating of objects with complex geometries and is compatible with a wide range of materials while requiring relatively low capital investment. An additional advantage is environmental friendliness due to the use of aqueous electrolytes. Limitations include the requirement for conductive or semiconductive substrates. Achieving uniform thickness and properties over large areas is challenging. Thick coatings may crack, and careful substrate cleaning and activation are required to ensure adhesion. Products of anodic dissolution may accumulate in the electrolyte, deteriorating coating quality. Not all materials can be deposited using this method [152,153,154,155,156,157,158]. The process is schematically shown in Figure 10.

#### 2.2.6. Thermal Spraying

**Figure 11:**
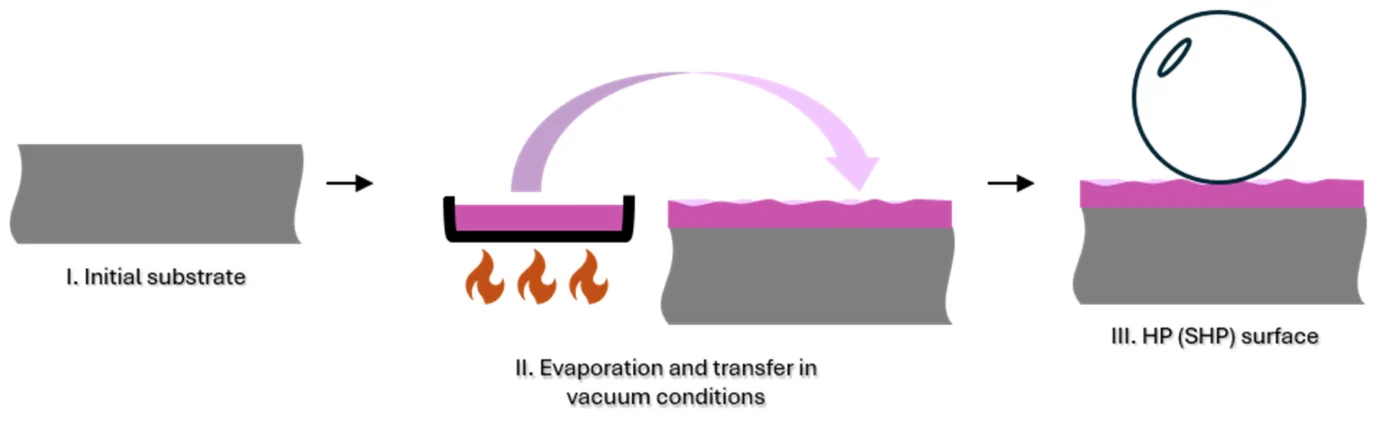
The scheme of thermal spraying. The flame icons indicate heating, while the pink arrow represents the transfer of material to the surface.

Thermal spraying is a vacuum deposition technique in which the source material is evaporated in vacuum and its vapor condenses on a substrate. Although this technology is not a primary method for producing hydrophobic and superhydrophobic coatings, it can be applied when specialized hydrophobizing precursors are used (Figure 11). Such precursors include fluorinated compounds (including certain high-temperature-stable fluoropolymers with inherent hydrophobicity), organosilicon compounds (organosilanes) that provide adhesion and water-repellent properties, and carbon nanomaterials (nanotubes, carbon black) that promote microstructure formation [159,160].

The key advantages of this method include high versatility (coating metals, ceramics, polymers, composites, concrete, brick, textiles, etc.), high deposition rate compared to some alternative techniques (sol–gel, electrodeposition, CVD, plasma treatment), and the possibility of precise thickness control using specialized equipment. However, the method has several limitations: difficulty in creating complex textured surfaces required for superhydrophobicity without additional processing (e.g., etching or templating), potential thermal damage to substrates, relatively low mechanical durability of thin films, porosity affecting corrosion resistance, and incompatibility with refractory materials or compounds that decompose below their evaporation temperature [159,160,161].

### 2.3. Control of Structure and Morphology at the Nanoscale to Realize HP and SHP Properties

Within this group of methods, the primary emphasis is placed on the high-precision fabrication of thin functional layers with nanoscale control over their composition and structure. This makes it possible not only to tailor the required physicochemical properties of the coating but also to deliberately create the hierarchical morphology necessary for the development of hydrophobicity and superhydrophobicity. The key methods and the quantitative characteristics used to evaluate the resulting coatings are presented below:

- Chemical vapor deposition (CVD) enables the deposition of dense or nanostructured coatings with high conformality, even on textured substrates. The main quantitative parameters include the coating thickness, which typically ranges from a few nanometers to several micrometers in multilayer deposition; porosity and density depend on the process temperature, gas mixture composition, and pressure; dense CVD coatings may exhibit porosity below 1–2%, whereas nanostructured coatings may reach 30–50% while maintaining mechanical integrity; the surface roughness (Ra, RMS) may vary from a few nanometers (for smooth dense films) to tens or hundreds of nanometers when a nanostructured morphology is formed; by using hydrophobic precursors (e.g., fluorinated compounds), contact angles of 140–160° can be achieved; adhesion to the substrate is evaluated by scratch testing or pull-off testing. Typical critical loads for the onset of coating delamination range from 15 to 30 N, depending on the substrate/coating combination [162,163];

- Self-assembly is based on the spontaneous organization of molecules or nanoparticles into ordered structures, thereby effectively reducing the surface energy. The quantitative parameters include the monolayer thickness, which is typically 1–3 nm for organic molecules (e.g., silanes or fluoropolymers); the molecular packing density is evaluated by ellipsometry and atomic force microscopy. A high packing density (close to the theoretical maximum) is required to minimize defects and achieve maximum hydrophobicity; for well-formed self-assembled layers, contact angles of 110–130° are typically obtained for hydrophobic systems and 150–170° for superhydrophobic systems, while contact angle hysteresis is also used as an important evaluation parameter; the coating stability is assessed by monitoring the retention of the contact angle after repeated wetting cycles, ultrasonic treatment, or exposure to aggressive environments [164,165,166];

- Layer-by-layer (LbL) deposition of oppositely charged components provides precise control over the coating thickness and composition. The important characteristics include the thickness of a single bilayer, which is typically 2–10 nm depending on the polyelectrolytes used and the deposition conditions; the total coating thickness is controlled by the number of deposition cycles and may range from tens of nanometers to several micrometers; the morphology and roughness are characterized by atomic force microscopy, which shows that the surface roughness (RMS) increases with the number of deposited layers and may reach 20–50 nm, leading to the formation of a hierarchical structure; the contact angle varies from 120 to 160° depending on the layer composition and the nature of the outermost layer (hydrophobic or hydrophilic); the mechanical stability is evaluated by cyclic testing and the tape test. Multilayer systems with properly selected polyelectrolytes retain their properties after 10–20 abrasion cycles [167,168,169,170];

- Electrospinning enables the fabrication of nanofibrous mats with high porosity and a well-developed hierarchical structure. The principal parameters include the fiber diameter, which typically ranges from 50 to 800 nm and depends on the solution viscosity, applied voltage, and the distance between the emitter and the collector; the mat porosity may reach 80–95%, promoting air entrapment and the development of the superhydrophobic effect; the mat thickness and fiber packing density are also important characteristics, with the layer thickness varying from tens of micrometers to 1–2 mm, while the packing density influences the mechanical strength and permeability; for nanofibrous mats fabricated from hydrophobic polymers (PVDF, PTFE, and their composites), contact angles of 150–165° are typically achieved, whereas the sliding angle may be lower than 10°; the mechanical properties, including tensile strength and elastic modulus, depend on the fiber orientation and the presence of binders; several studies have shown that the incorporation of nanoparticles (SiO_2_, ZnO) increases both the stiffness of the mat and its abrasion resistance [171,172,173].

#### 2.3.1. CVD

**Figure 12:**
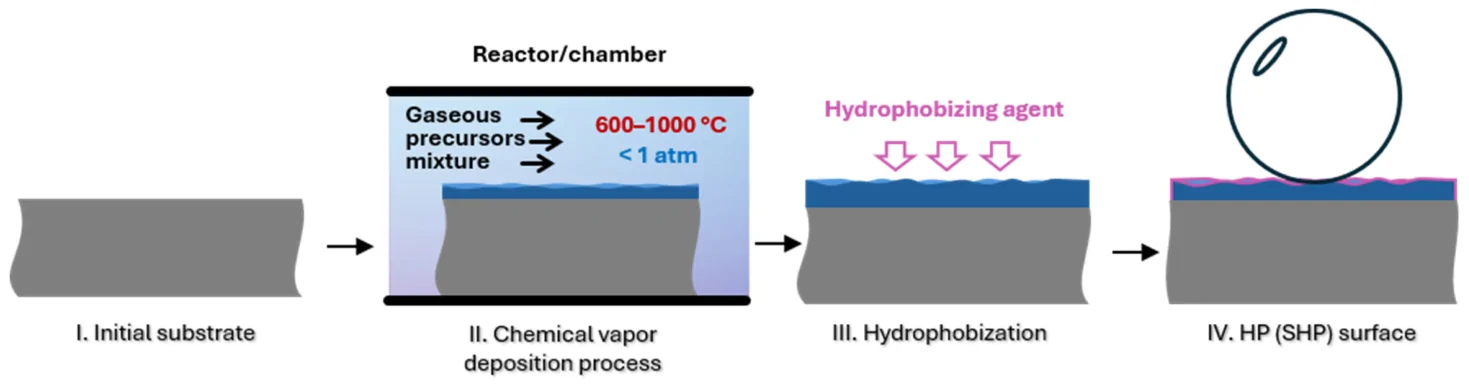
The scheme of CVD process. The different shades of blue represent the developed surface morphology, while the pink contour represents the hydrophobizing agent layer.

CVD is a process for forming solid coatings on a substrate through chemical reactions of precursors in the gas phase or directly on the substrate surface. Volatile by-products are removed by a carrier gas flow. HP and SHP coatings can be produced using various CVD techniques. The choice of a specific method depends on multiple factors, including required coating properties, substrate type, operating conditions, and economic considerations. To achieve hierarchical structures responsible for hydrophobic and superhydrophobic behavior via CVD, key process parameters must be controlled: temperature, pressure, precursor concentration, carrier gas flow rate, and deposition time. Variation in these parameters allows controlled tuning of nanoparticle size distribution and microstructure growth rate, enabling precise adjustment of surface morphology (Figure 12) [174,175].

The selection of precursors and reactive gases strongly influences hierarchical structure formation. For example, metal–organic compounds (β-diketonates, metal carbonyls, organometallic precursors of transition metals) or fluorinated compounds (perfluorocarbons, fluoropolymers, fluoroorganosilanes, fluorinated monomers) under appropriate reaction conditions (temperature, pressure, and gas-phase composition) promote the formation of multilevel micro- and nanostructures. Their decomposition behavior and interaction with the substrate enable the formation of coatings with tailored properties. Advantages of CVD include the ability to coat complex geometries, precise control of thickness and composition, strong adhesion to substrates, high material purity, and scalability for industrial applications. However, limitations include high operating temperatures in conventional CVD processes (600–1000 °C and above), restricting use with thermally sensitive materials (polymers, some alloys); toxicity of certain precursors; difficulty in controlling surface roughness; potential degradation of coatings upon exposure to water (e.g., hydrolysis of silanol groups); and relatively high equipment cost [176,177,178,179,180].

#### 2.3.2. Self-Assembly and LbL Deposition

**Figure 13:**
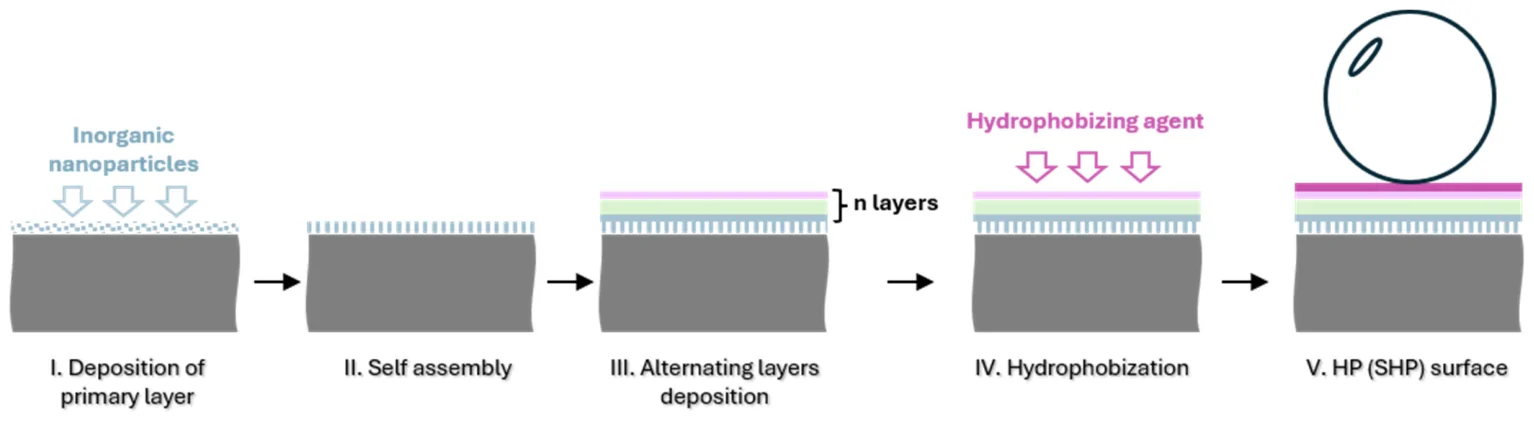
The scheme of self-assembly and LbL deposition. The blue-gray and white arrows indicate the deposition of inorganic nanoparticles. The blue-gray dots on the surface represent a disordered layer of these particles, while the blue-gray vertical bands represent an ordered layer. The horizontal lines represent layers of organic materials deposited using the layer-by-layer (LbL) technique. The magenta horizontal line in the right panel represents the hydrophobizing agent layer.

Self-assembly and LbL deposition is a method based on the formation of hierarchical surface structures combining chemical hydrophobicity and surface texturing. Initially, the surface is cleaned, and if necessary, etched or plasma-treated to create initial roughness or activate the surface to improve adhesion. A base layer is then deposited, typically consisting of inorganic nanoparticles (e.g., SiO_2_ or TiO_2_) or polymers applied by casting, spraying, or chemical deposition. In self-assembly processes, molecules or particles spontaneously organize on the surface due to intermolecular interactions such as van der Waals forces, hydrogen bonding, or hydrophobic interactions. In layer-by-layer deposition, alternating layers are sequentially applied—for example, polymer solution followed by nanoparticle suspension. A common approach involves alternating deposition of oppositely charged polyelectrolytes (e.g., polyallylamine hydrochloride, polystyrene sulfonate, etc.). Hydrophobizing agents (fluorosilanes, polytetrafluoroethylene, alkylsilanes) are then applied to the formed structure. Finally, thermal treatment, rinsing, or additional modification is performed to remove excess reagents, improve adhesion, and stabilize the coating (Figure 13) [181,182,183,184].

This method is applicable to a wide range of substrates, including metals (steel, copper, aluminum, etc.), semiconductors, polymers (PET, PVC, etc.), glass, ceramics, textiles, and porous materials. It enables flexible tuning of texture and composition, formation of hierarchical structures, and is technologically simpler than high-temperature methods. Although potentially scalable, industrial scale-up is limited by the risk of coating non-uniformity. However, this method has several limitations: complex control of the self-assembly process due to dependence on multiple parameters (concentration, temperature, pH), limited coating thickness (risk of cracking and delamination), low mechanical strength (brittleness and wear sensitivity), multistep processing increasing time and cost, scaling difficulties due to non-uniformity, and restrictions imposed by toxicity or high cost of some hydrophobizing agents (e.g., fluorinated compounds) [185,186,187].

#### 2.3.3. Electrospinning

**Figure 14:**
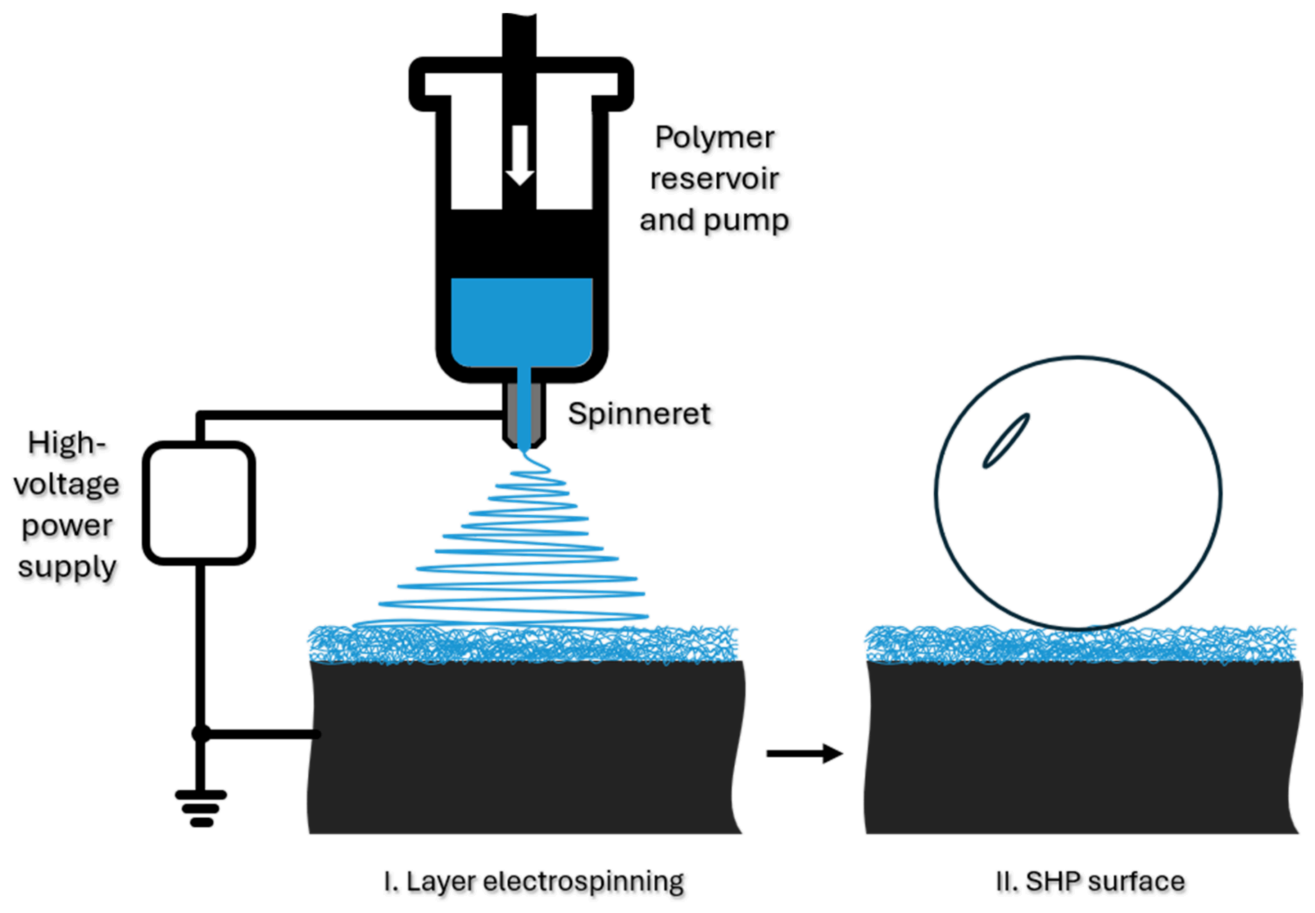
Generalized scheme of electrospinning.

Electrospinning is an electrohydrodynamic method for producing polymer fibers, in which a Taylor cone and a polymer jet are formed under an electric field, stretching into ultrafine fibers (10–1000 nm). The method enables the formation of highly porous structured coatings with hydrophobic and superhydrophobic properties due to the combination of low surface energy and controlled hierarchical micro- and nanostructures. Low surface energy is provided by inherently hydrophobic polymers (polytetrafluoroethylene/PTFE, fluoropolymers, modified polycaprolactone, polyurethane with fluorinated groups) or by introducing hydrophobizing agents into the electrospinning solution, such as perfluorinated compounds, stearic acid and its salts, organosilicon compounds (organosiloxanes, PDMS), and modified nanoparticles (SiO_2_, Al_2_O_3_). These additives migrate to the fiber surface, forming a hydrophobic layer (Figure 14) [5,188,189].

Hierarchical roughness is achieved by adjusting fiber diameter (through solution concentration, applied voltage, and feed rate), using coaxial electrospinning (“core–shell” structures where the shell contains hydrophobic components), or combining fibers of different diameters within a single coating. Advantages of the method include controllable morphology and coating thickness (regulated by voltage, polymer concentration, and other parameters), the ability to form hierarchical structures for superhydrophobicity, applicability to a wide range of polymers (including synthetic and biopolymers), and high porosity that improves adhesion and enables further surface modification. Disadvantages include a limited range of suitable polymers (particularly difficulties with poorly soluble or highly viscous materials), solvent toxicity and disposal issues, challenges in scaling up the process (reproducibility issues, needle clogging, fiber adhesion to collectors), high sensitivity to environmental conditions (temperature and humidity), low wear resistance of the resulting coatings compared to conventional methods, and the need for careful substrate pretreatment to ensure adhesion [19,190,191,192].

Overall, modern technologies for fabricating HP and SHP coatings demonstrate significant methodological diversity. Each technique has specific advantages and limitations that determine its suitability for particular applications. One of the key factors in selecting a fabrication method is the number of process stages, which directly affects production cost efficiency, technological complexity, final coating quality, and processing time (Table 2).

Thus, the selection of a fabrication technology should be based on a comprehensive analysis of the requirements for the final product, including substrate type, required functional properties, operating conditions, and economic factors. The most promising directions are hybrid (combined) technologies that integrate the advantages of different methods, as well as the development of new precursors and surface modification agents.

## 3. Materials for Hydrophobic and Superhydrophobic Coatings: Material Classes and Mechanisms of Coating Formation

Based on the data presented in Section 2, the following key classes of materials used for the fabrication of HP and SHP coatings can be identified: polymeric materials, inorganic compounds, and composite materials. The characteristics of the identified material groups are presented below.

### 3.1. Polymeric Materials

HP and SHP coatings based on polymeric materials are formed either by reducing the surface energy of the material or by creating a specific surface texture. Such polymeric materials include fluoropolymers (polytetrafluoroethylene, fluorinated organic compounds), silicon–organic polymers (silicones), polyurethanes, and polyacrylates [193,194,195,196,197,198,199,200,201,202,203,204,205,206,207,208,209].

#### 3.1.1. Fluoropolymers and Organofluorine Compounds

The fabrication of hydrophobic and superhydrophobic coatings based on fluoropolymers and organofluorine compounds is founded on a comprehensive strategy that combines control over both the chemical composition of the surface and its morphology. The desired properties are achieved through the sequential implementation of three interrelated technological stages, each governed by well-defined physicochemical mechanisms.

The first stage involves modification of the polymer surface layer, making it possible to deliberately reduce the surface energy of the material. This effect is achieved by replacing hydrogen atoms with fluorine atoms directly within the surface layer: the high electronegativity of fluorine and the formation of strong C–F bonds substantially reduce the surface polarity. The process is carried out using gaseous fluorine or other fluorinating agents (e.g., F_2_/He mixtures or CF_4_ plasma). The thickness of the fluorinated layer ranges from 10 nm to 10 µm and is controlled by the key technological parameters, including the pressure and composition of the gas atmosphere, temperature, and exposure time. The effectiveness of the modification is confirmed by an increase in the contact angle, as well as by Fourier-transform infrared (FTIR) spectroscopy and X-ray photoelectron spectroscopy (XPS), which reveal changes in the elemental composition of the surface layer [3,4,210,211].

The second stage involves the deposition of a thin fluoropolymer layer onto a previously structured (rough) surface. The optimum coating thickness is 2–8 nm. One of the most promising approaches is the use of supercritical carbon dioxide (scCO_2_) as a medium for dissolving and delivering fluoropolymer components; this method provides uniform coating even on complex profiles and microstructured surfaces without the defects characteristic of conventional solution-based techniques. The combination of the hierarchical surface morphology and the low surface energy of the deposited fluoropolymer makes it possible to achieve a water contact angle of 150° or higher, corresponding to the criterion for superhydrophobicity. The coating quality is evaluated by profilometry (Ra, Rq, and Sz parameters), scanning electron microscopy for morphological analysis, and measurements of the contact angle and sliding angle of water droplets [209,212].

The final stage is graft polymerization, which enables the formation of a coating with a controlled thickness and strong chemical adhesion to the substrate. Active sites capable of initiating polymer chain growth are first generated on the material surface using either radiation-based methods (γ-irradiation or electron-beam irradiation) or chemical methods (oxidative treatment or plasma activation). Fluorine-containing monomers (e.g., tetrafluoroethylene or hexafluoropropylene) are then grafted onto these active sites. The principal advantage of this method is the possibility of precisely controlling the coating structure while ensuring strong interfacial bonding through the formation of covalent bonds. The coating thickness and grafting degree are evaluated by ellipsometry, X-ray photoelectron spectroscopy, and thermogravimetric analysis [213,214,215].

Thus, the three-stage strategy makes it possible to independently control both the surface morphology and its energetic characteristics, which is critically important for the development of stable superhydrophobic properties. The combination of surface modification, deposition, and grafting techniques allows the technology to be adapted to a wide range of substrates and service requirements, as demonstrated by numerous studies on fluoropolymer coatings.

#### 3.1.2. Silicon–Organic Polymers

The fabrication of HP and SHP coatings based on organosilicon polymers is accomplished through a sequence of interconnected stages, each of which determines the final functional properties of the coating. The characteristics of these stages are presented below [104,192,206,207]:

- Chemical adsorption—silicon–organic compounds are physically adsorbed onto the substrate surface. At this stage, no strong chemical bonds are formed. Molecules of silicon–organic substances (polyorganosiloxanes, organosilanes, oligosiloxanes) orient themselves so that hydrophobic hydrocarbon radicals face the surrounding environment, while polar functional groups are oriented toward the substrate surface. This orientation reduces the surface energy of the system in accordance with the thermodynamic principle of free energy minimization. Depending on the nature of the substrate (e.g., the presence of hydroxyl groups on oxide surfaces, surface roughness, etc.), the degree of ordering of the layer may vary substantially [216,217];

- Formation of chemical bonds—after adsorption, chemical reactions occur in which reactive silane groups hydrolyze to form silanols (≡Si–OH), which then undergo polycondensation on the substrate surface forming ≡Si–O–Si≡ and bonds ≡Si–O–Me. As a result, a covalently bonded layer is formed where silicon–organic molecules are anchored to the surface. It should be noted that adsorption and bond formation often proceed simultaneously. Process control (humidity, temperature, exposure time) is required since hydrolysis and condensation are highly environment-sensitive [218,219,220];

- Optimization of HP properties—at the final stage, the molecular structure of the organosilicon compound plays a key role, particularly the length and nature of the hydrocarbon substituent attached to the silicon atom. Short alkyl chains (e.g., methyl, –CH_3_, or ethyl, –C_2_H_5_, groups) provide moderate hydrophobicity, with the contact angle (CA) typically ranging from 90° to 100°. To achieve pronounced hydrophobicity and, in particular, superhydrophobicity, compounds containing long alkyl chains (e.g., hexadecyl, –C_16_H_33_, or octadecyl, –C_18_H_37_) are employed, as they form a dense brush-like layer. Such packing of the hydrophobic tails minimizes the surface energy and prevents water from penetrating into the intermolecular space. As a result, the contact angle can reach 110–120° or higher, especially when the chemical modification is combined with the formation of micro- and nanoscale surface roughness (a hierarchical structure). The effectiveness of the surface modification is quantitatively evaluated by measuring the contact angle and sliding angle, performing profilometric analysis (surface roughness parameters R_a_, R_q_, and S_z_), and conducting coating stability tests in various environments (water, solvents, and repeated wetting cycles) [8,221,222].

Thus, the fabrication of hydrophobic and superhydrophobic coatings using organosilicon polymers represents a comprehensive technological sequence in which the processes of physical adsorption, chemical bond formation, and fine tuning of the surface layer structure are implemented successively. Careful control of the parameters at each stage—from the selection of an appropriate precursor to the optimization of the processing conditions (humidity, temperature, exposure time, and pH of the medium)—makes it possible to precisely tailor the performance characteristics of the resulting coating. In particular, the degree of hydrophobicity, the adhesion strength to the substrate, and the resistance of the coating to external influences can be deliberately controlled. The most effective strategy for achieving stable superhydrophobic properties is recognized as the synergy between two approaches: chemical surface modification with organosilicon compounds and the creation of hierarchical surface roughness. This combined approach not only substantially increases the contact angle but also ensures the long-term functionality of the coating under real service conditions. The effectiveness of this strategy has been repeatedly demonstrated in studies on surface chemistry and the development of advanced materials, making it highly promising for a wide range of practical applications [216,217,218,219,220,221,222].

#### 3.1.3. Polyurethanes

Several complementary approaches are employed for the fabrication of hydrophobic and superhydrophobic polyurethane-based coatings, enabling the surface properties of the material to be deliberately tailored [199,200,201,202,207,208]:

- Introduction of hydrophobic additives into the polymer composition—modification of the polyurethane matrix is achieved by incorporating hydrophobic components during the preparation of the polymer formulation, primarily organosilicon compounds (organosilanes, oligosiloxanes, and polysiloxanes), as well as fluorine-containing agents. During coating formation, the modifier molecules undergo thermodynamically driven migration toward the coating/environment interface: the polar functional groups are oriented toward the interior of the polymer matrix, whereas the hydrophobic moieties are exposed at the surface. As a result, a surface layer with reduced surface energy is formed, leading to decreased wettability and an increased CA. The effectiveness of this approach depends on the compatibility of the additive with the polyurethane matrix, the modifier concentration, and the kinetics of its migration to the surface [223,224];

- Creation of micro- or nanostructures—the surface morphology is of critical importance for achieving the superhydrophobic state (CA > 150°). This is accomplished by creating micro- or nanostructures that enhance water repellency through the Cassie–Baxter mechanism: the contact area between the water droplet and the solid surface is reduced, while the air layer trapped within the surface asperities stabilizes the non-wetting state. Practical approaches to surface structuring include the incorporation of fillers with controlled particle size and hydrophobically modified surfaces (e.g., SiO_2_, Al_2_O_3_, TiO_2_ particles). These particles generate the primary surface roughness and may additionally serve as crystallization centers or influence the curing kinetics. Surface relief can also be created using physicochemical texturing methods, including plasma etching, acid-alkali etching, lithography, laser microstructuring, and electrospinning. These techniques enable the formation of a hierarchical morphology (a combination of micro- and nanoscale features), which is particularly important for the stability of the superhydrophobic state. The surface morphology is characterized by scanning electron microscopy and atomic force microscopy, while the surface roughness is quantitatively evaluated by profilometry (R_a_, R_q_, and S_z_ parameters). The stability of the non-wetting state is confirmed by measuring the contact angle hysteresis and the sliding angle [8,17,225];

- Combined approach (chemical modification + surface texturing)—the most effective strategy for obtaining stable polyurethane-based superhydrophobic coatings is the combination of hydrophobic surface modification and surface texturing. During the first stage, the surface relief is created by incorporating particles or by surface treatment. During the second stage, a low-surface-energy layer is established through the migration or application of hydrophobic agents (fluorinated or organosilicon compounds). The synergistic effect arises from the simultaneous action of two factors: reduction in the water/solid contact area due to the surface relief (the Cassie–Baxter effect) and reduction in the surface energy at the microscale. This approach makes it possible to achieve contact angles exceeding 150° and sliding angles below 10°, while also improving the coating’s resistance to mechanical and environmental stresses [226,227,228].

#### 3.1.4. Polyacrylates

Polyacrylate systems also enable the implementation of various strategies for imparting hydrophobic and superhydrophobic properties [158,159,160,161,209]:

- Copolymerization with hydrophobic monomers—chemical modification of the polymer structure during synthesis by incorporating hydrophobic monomer units into the macromolecular chain. Monomers containing long alkyl or fluorinated substituents (e.g., lauryl methacrylate, stearyl methacrylate, and fluorinated acrylates) are introduced into the reaction mixture. During polymerization, copolymers are formed in which the hydrophobic fragments self-organize into microdomains with low surface energy. The critical concentration of the hydrophobic monomer required to achieve pronounced hydrophobicity is typically 10–30 wt.% of the total monomer mixture. The composition and structure of the copolymer are characterized by gel permeation chromatography (GPC), Fourier-transform infrared (FTIR) spectroscopy, and nuclear magnetic resonance (NMR) spectroscopy [229,230,231];

- Surface modification—in many cases, the optimal strategy is to modify only the surface layer of the polyacrylate coating, making it possible to tailor its functional properties without affecting the bulk characteristics of the material. This approach employs a combination of methods, including plasma treatment using low-temperature plasma of inert or functional gases, which regulates the surface energy and generates active sites for subsequent functionalization; application of hydrophobizing agents (including silane and fluoropolymer solutions), resulting in the formation of a thin low-surface-energy layer; and chemical grafting of hydrophobic monomers by radiation-induced or photochemical treatment, which ensures reliable fixation of the modified layer through the formation of covalent bonds. The depth of the surface modification and the detailed composition of the surface layer are characterized by X-ray photoelectron spectroscopy (XPS) and time-of-flight secondary ion mass spectrometry (ToF-SIMS), which together provide reliable characterization of the changes occurring within the near-surface region of the coating [232,233,234];

- Formation of textured surfaces—similarly to polyurethane systems, polyacrylate coatings can be structured by creating micro- and nanoscale surface relief through the incorporation of dispersed fillers (e.g., SiO_2_ and TiO_2_), lithography, etching, laser texturing, and electrospinning. Hierarchical surface roughness, combined with the inherently low surface energy of the polyacrylate matrix (or that of an additionally modified surface), enables the development of the superhydrophobic state. Particular attention is paid to preserving the optical properties of the coating (in the case of transparent polyacrylate systems) and the mechanical durability of the textured surface [8,235].

The choice of polymer material and coating formation method depends on target HP/SHP performance, operating conditions, and technological capabilities. The combination of chemical modification and surface texturing is the most promising approach for achieving SHP properties with CA > 150°.

### 3.2. Inorganic Compounds

HP and SHP coatings based on inorganic compounds (metal oxides SiO_2_ and Al_2_O_3_, SiO_2_ nanoparticles, carbides) are formed through a combination of surface structural features, chemical modification of materials, and the creation of hierarchical roughness. The key mechanisms include surface texturing, reduction in surface energy, and the use of hydrophobizing agents [85,236,237,238,239,240,241,242,243,244].

#### 3.2.1. HP and SHP Coatings Based on SiO_2_ and Al_2_O_3_ Oxides

The fabrication of hydrophobic and superhydrophobic coatings based on SiO_2_ and Al_2_O_3_ is based on a two-step strategy. First, a morphologically developed rough surface structure is created, after which the surface is chemically modified to reduce its surface energy. The combination of these two stages makes it possible to achieve CA values exceeding 150° and to impart self-cleaning properties to the coatings:

- Creation of a rough surface structure. Oxide surfaces are inherently hydrophilic because of the high concentration of surface hydroxyl (–OH) groups. To achieve superhydrophobicity, it is necessary to generate a micro- and nanoscale surface relief that stabilizes the non-wetting state through the Cassie–Baxter mechanism: the air layer trapped within the surface asperities minimizes the contact area between the water droplet and the solid surface. The following methods are commonly employed to create surface roughness: anodization, the sol–gel method, laser treatment, and etching [73,78,86,90,126];

- Chemical surface modification to reduce surface energy. Since SiO_2_ and Al_2_O_3_ surfaces are intrinsically hydrophilic, chemical modification is required to impart hydrophobic properties. This is achieved using hydrophobizing agents capable of forming strong covalent bonds with the hydroxyl groups of the oxide surface. Alkylsilanes (e.g., octadecyltrichlorosilane) form densely packed monolayers with long hydrocarbon chains; fluorosilanes (e.g., perfluorooctyltrichlorosilane) provide even lower surface energy owing to their fluorocarbon chains; polydimethylsiloxane is applied in the form of low-molecular-weight oligomers or solutions to produce flexible hydrophobic layers; and hexamethyldisilazane is used for surface silanization and passivation of the active –OH groups. The chemical modification process proceeds through three consecutive stages: chemisorption—the hydrophobizing agent (most commonly a chloro- or alkoxysilane) reacts with the surface hydroxyl groups of the oxide according to the reaction: ≡Si–OH + Cl–SiR_3_ → ≡Si–O–SiR_3_ + HCl. As a result, strong ≡Si–O–Si≡ bonds are formed, anchoring the modifier molecules to the surface. Formation of a covalently bonded monolayer—after the molecules are attached to the surface, intermolecular condensation of neighboring silane molecules occurs: Cl–SiR_3_ + HO–Si≡ → R_3_Si–O–Si≡ + HCl, leading to the formation of a cross-linked network and a stable monolayer. The hydrophobic moieties (hydrocarbon or fluorocarbon chains) are oriented outward, thereby creating a water-repellent barrier. Reduction in surface energy—the fluorocarbon or hydrocarbon chains provide a surface energy of approximately 10–20 mN/m, which is substantially lower than that of pristine oxides (70–80 mN/m). This results in a significant increase in the CA and the development of superhydrophobic properties [3,9,32,209,210,211,212,213,214,215,216,217,218,219,220].

#### 3.2.2. SiO_2_ Nanoparticles for the Formation of HP and SHP Coatings

When using silicon dioxide (SiO_2_) nanoparticles, the formation of HP and SHP coatings can be divided into the following stages:

- Formation of a rough structure from SiO_2_ nanoparticles. The initial SiO_2_ nanoparticles are hydrophilic, but their specific packing on the substrate creates the required micro- and nanoroughness for achieving HP and SHP properties. They can be deposited via solution dispersion, spraying, dipping, or spin coating. As a result, nanoparticles with diameters of 10–100 nm form nanoscale roughness, while their agglomerates and clusters create micro-protrusions with heights up to 60–100 µm [245,246,247];

- Surface hydrophobization—at this stage, chemical modification of SiO_2_ nanoparticle surfaces is carried out to reduce surface energy. The same hydrophobizing agents as those used for SiO_2_ and Al_2_O_3_ oxides are applied. They are introduced via drop-casting, immersion in solution, vapor-phase deposition, etc. The modification mechanism proceeds through the following stages: hydrolysis of the silane groups in the presence of trace amounts of moisture: Cl–SiR_3_ + H_2_O → HO–SiR_3_ + HCl; condensation with the surface –OH groups of the nanoparticles: HO–SiR_3_ + ≡Si–OH → R_3_Si–O–Si≡ + H_2_O. Intermolecular condensation of neighboring silane molecules, resulting in the formation of a cross-linked network. As a result, a covalently anchored monolayer is formed, in which the hydrophobic moieties are oriented outward [218,219,220,235];

- Formation of the HP or SHP state—the final characteristics of a hydrophobic or superhydrophobic coating are determined by the combined influence of the parameters established during the preceding stages of the technological process. The key governing factors include the degree of surface coverage by the hydrophobizing agent: insufficient coverage results in the formation of isolated hydrophilic regions that act as wetting centers and reduce the CA. Another critical factor is the packing density and degree of nanoparticle aggregation: an optimal configuration maximizes the fraction of air trapped within the surface relief, which is essential for stabilizing the non-wetting state. The thickness and uniformity of the hydrophobic layer also exert a significant influence. Excessive coating thickness often leads to the formation of multilayers with reduced long-term stability. Equally important are the mechanical properties of the coating: nanoparticle-based systems are frequently susceptible to abrasion, and therefore additional binders or thermal fixation of the coating layer are commonly employed to improve durability. A comprehensive set of methods is used for the quantitative evaluation of coating performance, including measurements of the CA and sliding angle, where CA values > 150° combined with sliding angles below 10° are considered reliable indicators of the superhydrophobic state; durability tests involving repeated wetting/drying cycles, water-jet exposure, abrasion testing, and UV irradiation to assess the retention of functional properties under service conditions; as well as analyses of the coating morphology and chemical composition [8,246,248].

#### 3.2.3. Formation of HP and SHP Coatings Based on Carbides

Carbide materials (e.g., SiC, TiC, WC, and B_4_C) are promising candidates for the fabrication of hydrophobic and superhydrophobic coatings owing to their combination of high hardness, chemical inertness, thermal stability, and ability to develop well-defined surface morphologies. The development of hydrophobic/superhydrophobic properties on carbide coatings is a multistage process in which each stage requires precise control of the processing parameters to achieve the desired performance characteristics:

- Formation of a carbide-based layer—carbide coatings are obtained using various methods such as CVD, PVD, solid-state diffusion methods, electrical discharge alloying, and laser modification. The selection of the fabrication method is determined by the coating requirements (thickness, porosity, and hardness), the type of substrate, and the availability of equipment. Quality control of the base layer is performed using X-ray diffraction (XRD) to confirm the phase composition, scanning electron microscopy (SEM) to evaluate the morphology, and nanoindentation to assess the mechanical properties [85,249];

- Formation of roughness—in carbides, roughness can be achieved in several ways: inherent roughness during carbide growth (during deposition or diffusion, surface irregularities, protrusions, or clusters may form, creating micro- and nanostructures), mechanical pre-treatment (e.g., sandblasting prior to coating deposition to enhance adhesion and create initial roughness), and post-deposition treatment (chemical or physical etching to increase surface roughness) [250,251];

- Chemical surface modification to reduce surface energy—even in the presence of roughness, carbide surfaces may remain hydrophilic due to polar groups or interactions with the environment. To impart HP and SHP properties, chemical modification is performed using fluorinated or silicon–organic compounds, including reactions with alkylsilanes or fluorosilanes that replace polar groups with hydrophobic ones, as well as the formation of composite structures where carbides are combined with hydrophobic nanoparticles (e.g., carbon nanotubes) or polymers that enhance water-repellent properties. Thus, the formation of HP and SHP coatings based on carbides is a multi-stage process requiring careful control of deposition parameters, surface treatment, and selection of compatible modification materials [8,218].

The formation of HP and SHP coatings based on inorganic compounds is a promising technological direction that offers broad opportunities for industrial implementation. Due to the unique functional properties of inorganic materials, high durability of the resulting coatings can be achieved, while their compatibility with modern deposition techniques ensures cost-effective solutions. Successful application of these technologies in various fields (aviation, energy, microelectronics, etc.) requires strict adherence to technological protocols and consideration of the specific characteristics of the processed materials.

### 3.3. Composite Materials

HP and SHP coatings based on composite materials utilize the advantages of a multicomponent structure. These include polymer nanocomposites, organometallic compounds, and carbon nanomaterials. The formation of the required coating properties occurs through the synergy of structural features, chemical interactions between components, and controlled surface morphology [252,253,254,255,256,257,258,259,260,261,262,263,264,265,266,267,268,269,270,271,272,273,274,275,276,277,278,279,280,281].

#### 3.3.1. Polymer-Based Nanocomposites for HP and SHP Coatings

The use of polymer-based nanocomposites leads to the formation of HP and SHP coatings due to the combination of a low-surface-energy polymer matrix and nanofillers that create surface roughness or modify surface properties. The mechanism of formation of such coatings can be divided into the following stages:

- Selection of the polymer matrix—hydrophobic polymers with nonpolar units are used to reduce surface wettability, such as polytetrafluoroethylene, polydimethylsiloxane, and certain polyacrylates. They provide a baseline level of hydrophobicity; however, the contact angle rarely exceeds 120° without additional modifications [257];

- Addition of nanofillers—the introduction of hydrophobic nanoparticles (silicon dioxide, titanium dioxide, carbon nanoparticles) into the matrix leads to the formation of micro- or nanoroughness. Increased surface roughness promotes the formation of air pockets between the droplet and the substrate, reducing the solid–liquid contact area and increasing the apparent contact angle, as described by the Wenzel and Cassie–Baxter models, thereby enhancing hydrophobicity [252,253,254];

- Chemical surface modification—to improve nanoparticle dispersion in the polymer and enhance hydrophobic properties, functionalization is performed (e.g., using silane agents or fluorinated compounds). This reduces the surface energy of the “nanoparticle–polymer” system, which is critical for achieving a contact angle > 150° and forming SHP surfaces [255,259];

- Creation of a hierarchical structure—the combination of micro- and nanoroughness (particles of different sizes) enhances the lotus effect (droplet roll-off) and promotes the Cassie–Baxter state (air trapping under the droplet), maximizing hydrophobicity [253,256];

- Stabilization of interfacial interactions—stabilizing additives or modifying agents (including grafted copolymers) prevent nanoparticle agglomeration and ensure uniform distribution within the matrix. This directly affects coating durability and mechanical strength: without such additives, nanoparticles tend to aggregate or be washed out, leading to structural degradation [257,258,259].

#### 3.3.2. Organometallic Compounds for the Formation of HP and SHP Coatings

Organometallic compounds are frequently used in the formation of HP and SHP coatings. The following mechanisms can be identified:

- Hydrolysis and condensation of organometallic compounds. This is the key chemical process underlying the sol–gel method. Organometallic precursors (e.g., silicon- or titanium-based compounds) undergo hydrolysis in the presence of water or water–alcohol mixtures, and the hydrolysis products subsequently condense to form a polymeric or oligomeric network. As a result, a gel is formed, which transforms into a solid coating upon drying. This mechanism determines the chemical structure and cohesion of the material [282,283];

- Formation of porous or rough structures. During hydrolysis and condensation, a developed surface morphology (micro- or nanoroughness) may form. Porosity and roughness increase the effective contact of the water droplet with air, reducing the solid–liquid contact area and enhancing hydrophobic properties. Hierarchical structures (combining different roughness scales) are particularly effective for achieving SHP behavior [284,285];

- Surface modification with hydrophobic groups. Hydrophobic moieties (e.g., fluorinated groups, alkyl radicals) may be introduced into the organometallic precursors or during post-treatment, reducing surface energy and decreasing water affinity [286,287];

- Stabilization of structure and improved adhesion. Long-term stability requires strong adhesion to the substrate and resistance to external influences. This is achieved by optimizing synthesis conditions (temperature, pH), using crosslinking agents, or incorporating additional components that improve bonding to the substrate. For example, during spin coating of precursor sols, it is important to control solution viscosity and rotation speed to ensure uniform deposition. Thermal treatment also influences the structure and hydrophobic properties of the coating [283].

#### 3.3.3. Carbon Nanomaterials for the Formation of HP and SHP Coatings

Carbon nanomaterials (graphene, carbon nanotubes, carbon black) are used for HP and SHP coatings due to their high specific surface area, aspect ratio, and ease of modification. The following mechanisms are realized:

- Creation of surface roughness. Carbon nanomaterials (e.g., nanotubes, nanofibers) can form a “forest-like” micro- and nanostructure. The voids between these elements are filled with air, reducing the water–solid contact area and enhancing hydrophobicity, known as the “lotus effect,” observed in nature (e.g., lotus leaves, reed plants, etc.) [270,274,275];

- Use of low-surface-energy materials. Carbon nanomaterials such as modified carbon nanotubes exhibit low surface energy, reducing wettability and promoting high contact angles [269,272,277];

- Surface modification with hydrophobic groups. Carbon nanomaterials can be treated with surfactants or hydrophobizing agents that orient on the outer surface of the coating, further reducing surface energy and enhancing hydrophobicity [271,278];

- Formation of composite structures. Carbon nanomaterials are often incorporated into composites with polymer matrices (e.g., silicone or epoxy). The polymer matrix provides adhesion and structural integrity, while carbon components provide hydrophobic and additional functional properties (e.g., electrical conductivity) [273,279,280];

- Deposition and processing methods. Deposition techniques (e.g., spraying, coating, xerogel deposition) and subsequent treatments (thermal treatment, drying, flame exposure) influence coating structure and properties. For instance, flame treatment can lead to the deposition of amorphous carbon nanoparticles forming SHP films [268,276,281].

Composite materials represent a promising basis for the development of modern HP and SHP coatings with tailored properties. Further development should focus on optimizing surface modification methods, creating more efficient stabilization systems, and designing innovative composite formulations capable of addressing specific application-driven requirements.

## 4. Durability of HP and SHP Coatings: Main Problems and Modern Methods of Ensuring It

### 4.1. Main Problems of Durability of HP and SHP Coatings

The durability of HP and SHP coatings is a complex issue associated with multiple factors affecting the preservation of their functional properties over time. Despite their advantages (water-repellent properties, protection against corrosion, biofouling, and icing), these coatings are often subject to degradation under the influence of environmental conditions, mechanical loads, chemical agents, and other factors.

A key challenge remains mechanical instability: the fragility of the micro- and nanostructures responsible for the superhydrophobic effect makes the coating vulnerable to abrasive impacts, including friction, mechanical shocks, and hydrodynamic erosion caused by raindrops. Damage to the textured structure results in the loss of essential functional characteristics, such as self-cleaning ability and water repellency. In particular, the impact force of droplets under intense rainfall or high-velocity water flows can cause significant degradation of the coating relief. Equally important is the chemical instability of the hydrophobic agent, which is primarily associated with the weak interaction between the hydrophobizing compound and the surface due to physical adsorption and hydrogen bonding. This may lead to non-uniform distribution of the modifier within the textured layer. Aggressive environments, including chemical reagents, detergents, organic solvents such as acetone and octane, as well as acidic and alkaline media, accelerate coating degradation [32,288,289,290].

Climatic factors also have a substantial impact, including temperature fluctuations, high humidity, freeze–thaw cycling, and extreme thermal loads. Particularly destructive is the sequential exposure to cycles of immersion, freezing, drying, and heating, which promotes material cracking and the propagation of microcracks. In addition, ultraviolet radiation accelerates degradation by breaking chemical bonds within the hydrophobic layer: prolonged and intense exposure to sunlight gradually deteriorates the coating properties. Thermal instability also limits service life: at elevated temperatures, partial volatilization of coating components may occur, while prolonged thermal exposure leads to evaporation of hydrophobic agents, making the use of thermally stable materials necessary to maintain functionality. Furthermore, durability is affected by technological limitations: coating defects and non-uniformity often arise due to improper processing, insufficient surface preparation, or inappropriate plasma treatment parameters, resulting in local structural imperfections [32,291,292,293,294].

Thus, the combined influence of the aforementioned factors (mechanical, chemical, climatic, thermal, and technological) significantly limits the practical application of hydrophobic and superhydrophobic coatings, despite their attractive functional characteristics.

### 4.2. Modern Methods to Increase the Durability of HP and SHP Coatings

Despite the wide range of functional advantages offered by hydrophobic and superhydrophobic coatings, their practical application is significantly limited by durability issues. As discussed in Section 4.1, the performance characteristics of these coatings deteriorate over time under the influence of multiple external factors, leading to degradation of the textured structure and loss of key functional properties. Therefore, the development of approaches for improving coating durability remains one of the priority research directions in surface engineering. The following modern strategies aimed at overcoming existing limitations and extending the service life of hydrophobic and superhydrophobic coatings are presented below:

- Laser treatment. The combination of laser texturing (which forms the microrelief) and chemical modification (which creates a protective layer) makes it possible to significantly improve the mechanical durability of the texture and the chemical stability of the coatings. In particular, laser texturing enables the formation of a surface microrelief providing a CA of up to 170.9 ± 0.7°. Additional UV–ozone treatment for 90 min allows the coating to withstand 1000 abrasion cycles, while the adhesion strength reaches 2–3 MPa. The effectiveness of this combined approach has been confirmed by numerous studies [22,23,24,295,296,297];

- Reinforcement of microstructures. This method ensures the preservation of hydrophobic properties even under significant mechanical loading. Experimental studies have demonstrated that reinforced microstructures retain a CA above 150° after substantial mechanical impacts, while the adhesion strength of the coating reaches 3.5 MPa [298,299,300]. The abrasion resistance of a hydrophobic Fe-containing amorphous coating has also been demonstrated, with preservation of its superhydrophobic properties after mechanical wear;

- Hierarchical structure. The presence of a hierarchical structure not only on the surface but also at a certain depth relative to the substrate allows hydrophobic and superhydrophobic properties to be maintained even after wear of the upper layer. According to [301], during hydrodynamic erosion tests involving high-velocity droplet impacts, superhydrophobic coatings with self-similar hierarchical structures retained CA values in the range of 160–165° even after 500 h of operation under aggressive conditions. This confirms the stability of the Cassie–Baxter state after damage to the surface layer. Data demonstrating the effectiveness of hierarchical structures in ensuring long-term stability of coating functional properties are reported in [76,302,303,304,305];

- Incorporation of a reinforced intermediate layer. The introduction of an adhesive or other reinforcing interlayer between the coating and the substrate improves adhesion strength to 3–4 MPa, resulting in a 40–50% increase in coating service life compared with conventional solutions [306]. In study [307], a “sandwich structure” concept was implemented using an intermediate epoxy resin layer specifically designed to strengthen the interfacial contact. Experimental results demonstrated an increase in adhesion strength to 3–4 MPa. The multilayer system maintained its key functional properties (superhydrophobicity and corrosion resistance) even under substantial mechanical loads. This was confirmed by tests involving 150 tape-peeling cycles and 80 abrasion cycles. The results of studies devoted to the influence of reinforced intermediate layers on the performance of hydrophobic and superhydrophobic coatings are presented in [250,308,309,310];

- Self-healing coatings. Such coatings possess the ability to regenerate damaged areas under the influence of external factors, particularly heat and moisture. Several studies have demonstrated the possibility of rapid recovery of the superhydrophobic state. For example, in systems based on PDMS and dynamic polymer networks, regeneration of damaged regions can be achieved within 2–3 min at temperatures of 30–40 °C due to thermally activated intermolecular interactions [311]. Retention of CA values above 155° has been confirmed in studies [312,313,314] through cyclic tests demonstrating repeated recovery of hydrophobicity after controlled damage;

- Optimization of the surface layer composition. An important direction for improving the durability of hydrophobic and superhydrophobic coatings is the targeted regulation of the morphology and chemical composition of the hydrophobic agent. Experimental evidence supporting the effectiveness of this approach includes the use of silicon dioxide nanoparticles and boron nitride nanotubes: such additives enable the achievement of CA values of 161–170° and increase coating wear resistance by 60% compared with baseline compositions [315,316]. The results of experimental and theoretical studies illustrating the influence of surface layer composition on coating functional properties are presented in [317,318,319];

- Thermal treatment. This method represents a technological approach for restoring the hydrophobic properties of coatings degraded by ultraviolet radiation. Under appropriate conditions, thermal treatment can effectively recover the water-repellent characteristics of the surface: the CA can be restored to 160–165°, while the stability of functional properties is maintained at temperatures up to 150 °C for 1000 h [320]. The effectiveness of thermal treatment as a method for extending the service life of hydrophobic and superhydrophobic coatings has been confirmed in a number of scientific publications [321,322,323].

Thus, modern approaches for improving the durability of hydrophobic and superhydrophobic coatings encompass a wide range of technological solutions, from modification of surface structures to targeted optimization of composition and implementation of functional self-healing mechanisms. The combination of several approaches often enables a synergistic effect, substantially extending coating service life while preserving their key performance characteristics under demanding operating conditions.

## 5. Analysis of Commercial Manufacturers of HP and SHP Coatings: Market, Environmental Aspects, Applications, and Prospects

### 5.1. The Market of HP and SHP Coatings

The modern market for HP and SHP coatings is actively developing, offering solutions for various industrial sectors. Understanding the practical applications of these technologies, their cost, and competitive advantages is critically important for assessing the potential for commercial use. Key market players include both international corporations and specialized companies offering innovative solutions in the field of HP and SHP coatings (Table 3).

The success of modern companies in the HP and SHP coating market is determined by their ability to balance innovation and economic efficiency. Market leaders already demonstrate the capability to develop products that not only exhibit high technical performance but are also accessible to end users. A key factor of competitiveness is a comprehensive customer-oriented approach that includes the development of customized solutions, technical support, and after-sales service.

Therefore, the HP and SHP coating market demonstrates strong growth potential, and its future development will largely depend on the ability of market participants to achieve an optimal balance between technological innovation, economic efficiency, and environmental sustainability.

### 5.2. The Ecological Footprint and Impact of the Production of HP and SHP Coatings

The production of hydrophobic and superhydrophobic coatings is associated with a number of environmental impacts that must be considered at all stages of the product life cycle. The environmental footprint is significantly influenced by the selection of raw materials, the energy intensity of technological processes, and the use of potentially hazardous reagents. A substantial proportion of conventional hydrophobic and superhydrophobic coatings is based on organofluorine compounds, which are characterized by high resistance to degradation and a tendency toward bioaccumulation. Their presence in coating formulations creates long-term risks for ecosystems. In addition, several methods used for the formation of textured surfaces (e.g., chemical etching and anodization) are energy-intensive and require the use of aggressive chemical reagents, thereby increasing the environmental burden during the manufacturing stage.

Another important factor is the use of organic solvents (ethanol, toluene, acetone, etc.) during the application of certain types of coatings. Under large-scale production conditions, such substances may significantly increase emissions of volatile organic compounds (VOCs), negatively affecting air quality and occupational health [129,157,333].

At the same time, hydrophobic and superhydrophobic coatings can provide positive environmental effects by extending the service life of materials and reducing the need for resource-intensive maintenance operations. For example, in the textile industry, hydrophobic treatment can reduce the frequency of washing, thereby saving water and detergents. In the construction sector, protective coatings for facades reduce the need for regular maintenance and repair, leading to a decrease in the volume of construction waste. Similarly, ceramic hydrophobic coatings reduce the need for toxic chemicals used for protection against moisture, mold, and corrosion [76,334,335,336].

An additional advantage of modern approaches is the possibility of integrating functional additives that provide self-cleaning properties. For instance, the incorporation of photocatalytic components into coating formulations (e.g., titanium dioxide in the anatase modification) enables the degradation of organic contaminants and the transformation of toxic compounds into less harmful products under ultraviolet irradiation. This reduces the demand for chemical cleaning procedures and decreases anthropogenic pressure on the environment. Nevertheless, degradation of hydrophobic and superhydrophobic coatings under the influence of external factors (ultraviolet radiation, mechanical abrasion, and aggressive environments) gradually leads to deterioration of their functional properties. The need for periodic coating renewal generates an additional environmental footprint associated with repeated consumption of resources and waste generation [337,338].

Thus, the environmental assessment of hydrophobic and superhydrophobic coatings should consider not only direct emissions and resource consumption during the manufacturing stage but also indirect effects associated with the service life and operational performance of the coatings.

### 5.3. Promising “Green” Technologies and Environmentally Friendly Approaches in the Development of HP and SHP Coatings

Modern research in the field of hydrophobic and superhydrophobic coatings is increasingly focused on the development of environmentally friendly solutions aimed at minimizing negative impacts on the environment. A key direction is the replacement of toxic components, primarily organofluorine compounds and biocides, with safer and biodegradable alternatives. One of the most promising approaches involves the use of natural polymers and their derivatives. Plant-based waxes, modified cellulose, and various biopolymers are being considered as the basis for hydrophobic coatings. Such materials exhibit reduced toxicity and biodegradability, which significantly decreases their environmental footprint compared with conventional fluorine-containing formulations. In parallel, coatings based on organosilicon compounds are being actively developed, as they are characterized by lower environmental hazards while maintaining high hydrophobic performance.

Particular interest is focused on the development of environmentally friendly antifouling coatings for marine transport. Previously, antifouling formulations containing toxic biocides (tin, copper, and nickel salts) were widely used to prevent biofouling of ship hulls. These compounds are capable of accumulating in aquatic ecosystems and being transferred through food chains. Modern approaches are aimed at developing coatings that prevent the adhesion of marine organisms through low surface energy, without the use of biocidal components. This strategy makes it possible to effectively address the problem of biofouling while avoiding harmful effects on marine flora and fauna [339,340,341,342].

An important research direction is the development of composite materials with a reduced carbon footprint. This involves not only the selection of low-carbon raw materials but also the optimization of technological processes to reduce energy consumption and greenhouse gas emissions. In particular, one-step coating deposition methods are being investigated, allowing the number of manufacturing operations to be reduced and, consequently, lowering the overall carbon footprint of the products. A promising trend is the integration of hydrophobic and superhydrophobic coatings with digital technologies to improve their environmental efficiency. The application of IoT sensors for monitoring coating condition enables optimization of maintenance and repair schedules, preventing premature replacement of materials. This contributes to waste reduction and more efficient resource utilization [343,344,345,346,347].

Thus, the development of “green” technologies in the field of hydrophobic and superhydrophobic coatings is aimed at creating safe, biodegradable, and resource-efficient solutions that comply with the principles of sustainable development and minimize negative environmental impacts.

### 5.4. Current Research Objectives and Prospects for the Development of the HP and SHP Coating Industry

Modern science and industry are facing a number of key challenges that determine the further development of hydrophobic and superhydrophobic coating technologies. One of the priority objectives remains the development of new hydrophobic agents with improved operational characteristics, including enhanced resistance to external influences, extended service life, and reduced toxicity. Particular attention is being devoted to the search for formulations free of organofluorine components, in accordance with global environmental trends [348,349,350,351].

The improvement of methods for controlling micro- and nanostructures responsible for the formation of hierarchical surface morphology is also of significant importance. Precise control over relief parameters (height, spacing, and density of structural elements) directly affects the achievement of the required contact and sliding angles, as well as the resistance of coatings to mechanical and climatic influences. The development of high-precision texturing methods, including laser treatment, plasma modification, and electrochemical techniques, makes it possible to purposefully create structures providing stable hydrophobic and superhydrophobic properties [352,353,354].

Another important challenge is the investigation of the influence of various environmental and operational factors on the durability of hydrophobic and superhydrophobic coatings. In particular, the effects of ultraviolet radiation, abrasive wear, temperature fluctuations, and aggressive chemical environments on the stability of coating functional properties are being studied. The obtained data enable prediction of coating service life under different operating conditions and the development of strategies for improving their durability [355,356,357,358].

In the future, the implementation of several innovative solutions is expected, which may fundamentally improve the characteristics of hydrophobic and superhydrophobic coatings and expand their application areas. One of the most promising directions is the development of self-healing coatings based on microcapsules or reversible chemical bonds. Such coatings are capable of restoring hydrophobic properties after damage or contamination, significantly increasing their service life. It is predicted that the application of self-healing technologies will increase coating durability by 30–50% compared with existing solutions. Optimization of manufacturing processes represents another important development direction. The introduction of energy-efficient deposition methods, reduction in the number of technological operations, and transition to less toxic reagents may reduce production costs by 15–25%. This will make hydrophobic and superhydrophobic coatings more accessible to a wider range of industrial consumers and accelerate their implementation across various sectors [359,360,361,362].

The expansion of application areas for hydrophobic and superhydrophobic coatings also remains one of the key future prospects. In addition to traditional fields (construction, textiles, and transportation), active implementation of such coatings is expected in the energy sector (protection of solar panels against contamination), medicine (antibacterial and anti-adhesive surfaces), and microelectronics (moisture protection of components). The development of multifunctional coatings combining hydrophobicity with additional properties, such as self-cleaning ability, catalytic activity, and electrical conductivity, opens new opportunities for the creation of advanced functional materials [363,364].

Thus, current research challenges and future development trends in hydrophobic and superhydrophobic coatings are focused on improving their operational performance, reducing manufacturing costs, and expanding their functional capabilities. The successful implementation of these approaches will not only improve existing solutions but also create new markets for hydrophobic and superhydrophobic technologies.

## 6. Conclusions

Hydrophobic and superhydrophobic coatings represent one of the rapidly developing areas of modern surface engineering due to their ability to provide a combination of functional properties, including water repellency, self-cleaning behavior, anti-icing protection, and mitigation of corrosion and biofouling. The conducted analysis demonstrated that hydrophobic and superhydrophobic states can be achieved using a wide range of polymeric, inorganic, and composite materials and through diverse fabrication technologies. Despite differences in composition and preparation routes, the most effective approaches generally rely on the combination of hierarchical micro- and nanostructured surface topographies with reduced surface energy.

The review shows that the performance of hydrophobic and superhydrophobic coatings can be substantially improved through targeted control of surface morphology, coating composition, and interfacial structure. Optimized systems based on hierarchical structures have demonstrated contact angles of 160–165° after up to 500 h of hydrodynamic erosion. The incorporation of reinforced intermediate layers can increase adhesion strength to 3–4 MPa and extend coating service life by 40–50%. In addition, the incorporation of SiO_2_ nanoparticles and boron nitride nanotubes has been reported to provide contact angles of 161–170° while increasing wear resistance by up to 60%. These results demonstrate that achieving high initial hydrophobicity is not sufficient for practical applications and that the long-term preservation of surface morphology, chemical composition, and adhesion is a key requirement for technological implementation.

The analysis also indicates a gradual transition of hydrophobic and superhydrophobic coatings from predominantly laboratory-scale developments toward practical industrial applications. Nevertheless, insufficient durability, together with environmental concerns associated with persistent fluorinated compounds and the cost and complexity of some fabrication processes, remains among the main barriers to widespread implementation. Promising directions include the development of self-healing and regenerable coatings, fluorine-free hydrophobic formulations, more precisely controlled hierarchical structures, reinforced multilayer architectures, and scalable and energy-efficient fabrication technologies. In particular, self-healing systems capable of restoring superhydrophobic properties within several minutes and maintaining contact angles above 155° after repeated damage demonstrate the potential of active approaches to extending coating service life. Further integration of structural, chemical, and self-healing strategies is expected to enable the development of durable, multifunctional, and environmentally sustainable hydrophobic and superhydrophobic coatings for a broader range of industrial applications.

## Figures and Tables

**Table 1:** Functional characteristics of hydrophobic and superhydrophobic coatings.

Property	Description	References
Water repellency	Water droplets form nearly spherical beads and roll off the surface, minimizing contact with the material	[16,52,53,54]
Self-cleaning ability	Droplet mobility and reduced contaminant adhesion facilitate the removal of dust, dirt, and other hydrophilic particles	[28,29,30,35]
Anti-icing properties	Low ice adhesion delays ice accumulation and facilitates ice removal from the surface	[22,25,33,34,43]
Corrosion resistance	By limiting the contact of the substrate with water and oxygen, the coating reduces the risk of corrosion	[14,35,36,37,47]
Biocidal activity	Coatings containing active agents (e.g., silver ions, copper ions, or biocidal compounds) suppress the growth and proliferation of microorganisms	[48,49,50]
Drag reduction in liquid flows	In certain cases, hydrophobic surfaces can reduce hydraulic resistance by decreasing the liquid–solid contact area. However, the magnitude of this effect depends on surface roughness and other factors	[55,56,57,58]
Resistance to water droplet erosion	Surface texture and trapped air pockets can mitigate the impact of liquid droplets, thereby reducing erosion damage	[38,39,40]
Antifouling properties	Superhydrophobic coatings may inhibit the attachment of microorganisms and other fouling organisms, particularly when combined with biocidal additives	[44,45,46,47]

**Table 2:** Technological features of HP and SHP coating fabrication: process staging.

Method	Process Staging
Etching	Multistep
Anodization	Multistep
Laser treatment	Multistep
Lithography	Multistep
Plasma treatment	One-step
Hydrothermal synthesis	Multistep
Sol–gel	Combined. Depending on the specific variant of the method, one-step coating formation may be possible
Wet chemical reaction	Combined. Formation of HP and SHP coatings can proceed either in a single step or multiple steps due to the flexibility of the process: in the one-step route, hydrophobizing agents are introduced directly into the precursor solution, whereas in the multistep route they are applied as a separate post-treatment step
Dip coating	Combined. Proper selection of polymer components and fillers enables one-step coating deposition without the need for additional surface roughening
Spray coating	Combined. In some cases, a two-layer coating approach is applied to mimic the “lotus effect”: first, a hydrophobic base layer is formed via deposition of hydrophobizing agents; then, a suspension or dispersion of particles/powders is deposited onto the base layer to create hierarchical roughness, thereby converting a hydrophobic coating into a superhydrophobic one
Electrodeposition	Combined. A one-step process can be achieved through the synergistic action of electrolyte components (e.g., cerium nitrate and hexadecanoic acid), which simultaneously form hierarchical surface texture and reduce surface energy
Thermal spraying	One-step
CVD	Combined. The CVD method enables the formation of micro- and nanostructures on the surface, while subsequent treatment with hydrophobizing agents provides hydrophobic and superhydrophobic properties. However, under properly selected precursors, one-step fabrication of HP and SHP coatings is also possible
Self-assembly and Layer-by-layer	Multistep
Electrospinning	One-step

**Table 3:** Comparative characteristics of commercial manufacturers in the field of HP and SHP coatings.

Company	Description	Application Industries	Competitive Advantages	Coating Performance Support	Ref.
PPG	Offers protective coatings including HP properties, especially in the High Performance Coatings line. These coatings are designed to protect steel, concrete, and other materials from atmospheric effects and corrosion damage	Infrastructure, mining industry, oil and gas sector, energy, water treatment	Wide range of coatings for different substrates, technological adaptability, customization for specific tasks, systems with improved resistance to aggressive environments	Regular monitoring of coating condition, compliance with operating conditions (temperature, humidity), timely recoating upon wear	[324]
3M	Developed the “3M methodology” (materials, methods, morphology) for creating SHP materials based on polydimethylsiloxane	Marine industry, antifouling technologies	Use of nanotechnology to achieve SHP properties, adaptability to different substrates and operating conditions	Protection from mechanical damage, control of UV radiation and chemical exposure	[325]
NEI Corporation	Produces NANOMYTE^®^ SHP coatings, including SuperCN, SuperCN Plus, and WaterOFF. The first two are thermally cured, durable, transparent, highly hydrophobic coatings initially developed for metals but also applicable to plastics, glass, painted surfaces, and textiles. The third is a durable, fully transparent water-repellent coating for glass improving visibility in rain, snow, and slush and facilitating removal of ice, salt, dirt, and insects	Industry (condensers, industrial condensers), household appliances (kitchen devices, shower cabins), automotive industry	Resistance to abrasion, erosion, UV radiation, and chemicals; ability to customize for specific applications	Compliance with application and operating conditions, periodic inspection of coating integrity	[326]
BASF	Produces water-based hydrophobizing agents for protection of concrete surfaces from aggressive atmospheric exposure. These agents prevent capillary water absorption, improve resistance to weathering, maintain vapor permeability and durability, and are resistant to UV radiation and efflorescence	Construction (concrete, brick, stone facades), road surfaces, hydraulic structures, architectural elements	Deep penetration into mineral substrates, long-term moisture protection, preservation of natural surface appearance, reduced risk of freeze–thaw cracking	Proper application on prepared surfaces, compliance with technological standards	[327]
Ant Lab	Develops HP coatings, including HYDRAF food-safe nanocoatings. Designed to reduce liquid loss in the food industry by minimizing liquid adhesion to container surfaces	Food industry	Food-contact safety (FDA compliant), wear resistance, easy application, surface cleaning with a wet cloth without strong chemicals	Regular cleaning without aggressive agents, monitoring of mechanical damage	[328]
Surfactis	Develops SHP, durable, and semi-transparent coatings with self-cleaning, water-repellent, and anti-icing properties	Construction, energy, railway industry, watchmaking, optics	High performance, wear resistance, transparency	Protection from extreme mechanical and chemical exposure, UV radiation, regular visual inspection	[329]
Zirax	Provides hydrophobizing agents used in drilling fluids for oil and gas well operations. These agents are compatible with various saline solutions, resistant to mineral aggression, and stable over a wide temperature range	Oil and gas industry	Compatibility with saline systems, resistance to mineral aggression, stability across wide temperature ranges	Compliance with usage conditions in drilling fluid systems	[330]
BK-HIM	Produces and applies HP protective coatings for stone, wood, metal, and other construction materials. Based on organosilicon and fluorinated compounds, forming a film that protects surfaces from water, dirt, UV radiation, leaching, and fungal growth	Construction	Protection against a wide range of external factors, long durability	Regular inspection, compliance with operating conditions	[331]
ATF	Specializes in biocide-free antifouling coatings based on hydrophobicity. These coatings prevent biological fouling in oil and gas equipment and exhibit anticorrosion, antifriction, and chemical resistance properties. Applied by spraying, forming a thin dry film strongly adhered to the substrate	Oil and gas industry	Effectiveness against biofouling, compatibility with oil and gas equipment	Regular inspection, compliance with operating conditions	[332]

## Data Availability

No new data were created or analyzed in this study. Data sharing is not applicable to this article.
